# DPubChem: a web tool for QSAR modeling and high-throughput virtual screening

**DOI:** 10.1038/s41598-018-27495-x

**Published:** 2018-06-14

**Authors:** Othman Soufan, Wail Ba-alawi, Arturo Magana-Mora, Magbubah Essack, Vladimir B. Bajic

**Affiliations:** 1 Institute of Parasitology, McGill University, Montreal, QC H9X 3V9 Canada; 2 Princess Margaret Cancer Centre, University Health Network, Toronto, ON M5G 1L7 Canada; 3 Department of Medical Biophysics, University of Toronto, Toronto, ON M5G 1L7 Canada; 4 Computational Bio Big-Data Open Innovation Laboratory (CBBD-OIL), National Institute of Advanced Industrial Science and Technology (AIST), Tokyo, 135-0064 Japan; 5 Computational Bioscience Research Center, King Abdullah University of Science and Technology (KAUST), Thuwal, 23955-6900 Saudi Arabia

## Abstract

High-throughput screening (HTS) performs the experimental testing of a large number of chemical compounds aiming to identify those active in the considered assay. Alternatively, faster and cheaper methods of large-scale virtual screening are performed computationally through quantitative structure-activity relationship (QSAR) models. However, the vast amount of available HTS heterogeneous data and the imbalanced ratio of active to inactive compounds in an assay make this a challenging problem. Although different QSAR models have been proposed, they have certain limitations, e.g., high false positive rates, complicated user interface, and limited utilization options. Therefore, we developed DPubChem, a novel web tool for deriving QSAR models that implement the state-of-the-art machine-learning techniques to enhance the precision of the models and enable efficient analyses of experiments from PubChem BioAssay database. DPubChem also has a simple interface that provides various options to users. DPubChem predicted active compounds for 300 datasets with an average geometric mean and *F*_1_ score of 76.68% and 76.53%, respectively. Furthermore, DPubChem builds interaction networks that highlight novel predicted links between chemical compounds and biological assays. Using such a network, DPubChem successfully suggested a novel drug for the Niemann-Pick type C disease. DPubChem is freely available at www.cbrc.kaust.edu.sa/dpubchem.

## Introduction

Comprehensive and expanding public resources, such as the PubChem BioAssay Database (BioAssayDB)^1^, provide access to biological activity information from high-throughput screening (HTS) experiments. The vast amount of available data allows for the development of quantitative structure-activity relationship (QSAR) models to predict biological activities of chemical compounds for individual assays, enabling the so-called virtual (*in silico*) screening. QSAR models for virtual screening are derived by the standard ligand-based computational technique used in drug discovery to examine the compound libraries and to find potential candidates for binding with a specific and known biological target^2–4^. From a computational perspective, virtual screening involves analysis of large amounts of input data, integration of heterogeneous types of data, different statistical measures, and reliable selection of unbiased significant results and predictions^2,5,6^. These challenges, unless addressed in a carefully designed computational setup, cannot be carried out efficiently in later experimental phases in the process of drug discovery. Although several QSAR models implemented as web tools for predicting chemical-protein interactions have been developed^7–15^, they are limited in many aspects, for example, prediction performance is hampered by the imbalanced data in the HTS assays (the number of active compounds is usually significantly smaller than the inactive), the type of models available to the user as well as the flexibility to tune their parameters. Therefore, tools that possess the ability to reduce these limitations are of interest. Here, we introduce Dragon PubChem (DPubChem), a novel web tool to derive QSAR models for virtual screening of biological activity of chemical compounds. DPubChem reduces some of the limitations mentioned above by offering a rich set of options to the user that are easy to choose from (2 input types $\times$ 6 types of chemical features $\times$ 6 feature selection methods $\times$ 7 solutions for addressing class imbalance $\times$ 7 types of classifiers). Moreover, by considering the correlation of the HTS data, DPubChem allows for multi-label learning, where several HTS assays may be simultaneously used to derive QSAR models for more enriched virtual screening tasks. Since all the different options available in DPubChem tool are easy to use, it is straightforward to run several experiments and compare different models in order to select the optimal model. The results obtained from the 300 selected datasets, composed of 116,751 interactions and characterized by high class imbalance data, show that DPubChem is able to outperform existing QSAR models and that it achieved an average geometric mean (GMean) and *F*_1_ score (referred as *F*_1_, hereafter) of 76.68% and 76.53%, respectively. Half of the considered 300 datasets represent bioassays with hundreds to thousands of chemical compounds. Nevertheless, other datasets with a fewer number of compounds were also included to show the general applicability of DPubChem and to demonstrate that the implemented recognition models in the tool do not require large datasets for deriving robust models (as opposed to other recognition models that normally require large training data, such as deep learning models^16,17^). To the best of our knowledge, DPubChem is the only tool that provides: 1) an efficient mechanism to retrieve and analyze PubChem BioAssays, 2) an implementation of state-of-the-art machine learning algorithms (i.e., class imbalance and multi-label methods) to build QSAR models, and 3) a tool to rank and visualize unknown activity predictions for hundreds of chemical compounds provided by the user. DPubChem aims to provide an easy to use tool that will help biologists, biochemists, and experimentalists obtain useful insights about the chemicals and drugs of interest.

## Results

The key contribution of our study is the development of DPubChem, a novel and freely available web tool for deriving QSAR models for virtual screening of biologically active compounds from PubChem assays. The DPubChem tool implements the state-of-the-art methods for mining HTS data and provides users with an extensive but easy to use set of options to build robust models without compromising the simplicity of the interface.

In this section, we compare DPubChem to existing tools and provide an overview of its interface. We then show the results obtained when using the-state-of-the-art methods for multi-label classification (MLC) and for addressing the data class imbalance. Finally, we provide a case study analysis where we used DPubChem to suggest a drug for the Niemann-Pick type C (NPC) disease.

### Comparisons with other web servers and interface overview

Compared to many existing web servers for 3D docking^18–22^, which rely on ligand-protein docking, a smaller number of data-driven online systems were developed for virtual screening of chemical activities. As opposed to the 3D docking servers, the data-driven approaches do not require any prior knowledge of 3D structures of the target and its ligand. In addition, when data-driven models are trained, they can be used for screening the biological activity status of a set of chemicals faster than ligand-protein docking approaches^23^, which is an issue in screening a large number of compounds.

There are several web tools for predicting chemical-protein interactions^8,10,15^. The OCHEM^14^ and ChemBench^12,13^ are among the first freely available tools to mine HTS assays and allow users to derive different prediction models based on the user’s input. However, these tools require several data processing steps to produce a predictive model, and these may not be straightforward for the user. The HitPick^10^ tool has a simpler interface with a fixed model based on 2D molecular fingerprints and a Laplacian-modified naïve Bayes classifier. Later, the STITCH tool^7–9^ was developed to facilitate the search for the interactions of chemicals and proteins from a unified database extracted from different databases and literature. However, the main aim of STITCH is to provide a comprehensive database not specifically for the development of QSAR models. The tools mentioned above have certain limitations, for example, they do not address the imbalanced data of HTS assays and thus, are unable to reduce the false positive predictions. Moreover, some of these tools offer the flexibility to select different types of prediction models and parameters but at the expense of the simplicity. Finally, some of these tools also lack integration with a chemical compounds database. With all these shortcomings in mind, DPubChem tool focuses on the usage simplicity, flexibility, and prediction performance. Table 1 summarizes the characteristics of the QSAR tools mentioned above.

**Table 1:** Characteristics of the virtual screening tools. Note: SDF refers to the structure files which store the structural information of one or more compounds in a dataset.

Tool	ChemBench^12,13^	OCHEM^14^	HitPick^10^	MTI-OpenScreen^11^	DPubChem
Approach	HTS assay mining	HTS assay mining	HTS assay mining	Docking based screening	HTS assay mining
Input data type	SDF	SMILES, MOL2, SDF	SMILES	MOL2, SDF	SMILES, PubChem CID, BioAssay ID
Prediction model configuration	Flexible	Flexible	Fixed	Fixed	Flexible
Activity prediction (# of screening chemicals)	Yes (unlimited)	Yes	Yes (100)	Yes (5,000)	Yes (unlimited)
Addressing class imbalance or advanced preprocessing	No	No	Yes	No	Yes
Network visualization	No	No	No	No	Yes

MOL2 is a file containing the information to reconstruct a SYBYL molecule. SMILES stands for simplified molecular input line entry system and is a string of characters that represents a molecule. PubChem CID is a non-zero accession number representing a unique chemical structure.

**Figure 1:**
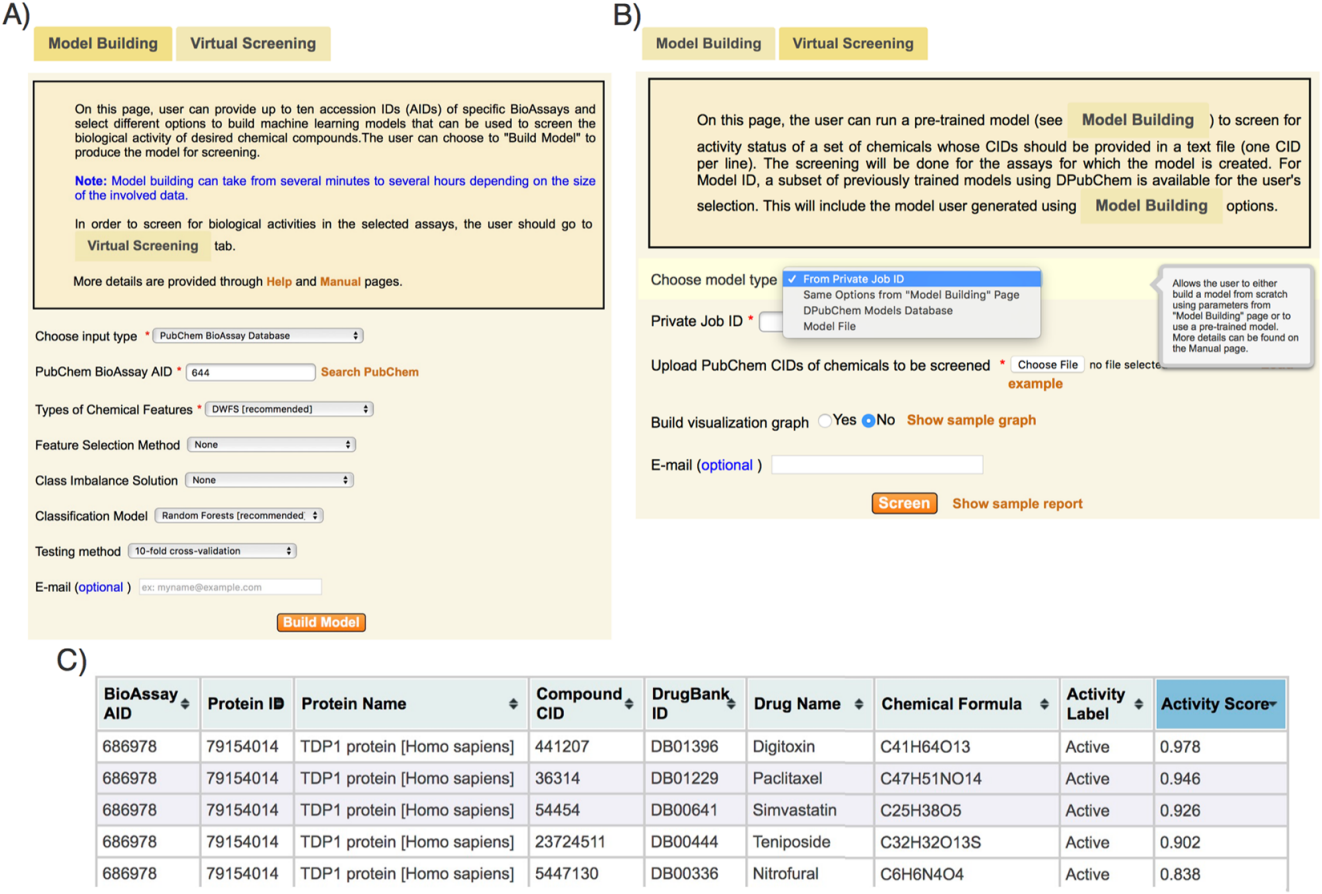
(**A**) Screenshot of the Model Building page in DPubChem. Several options are available to build different types of machine learning models to predict biological activities of provided chemical compounds. (**B**) Screenshot of the DPubChem virtual Screening page. The user can simply provide Job ID of a previously trained model and submit a list of compounds for activity screening. (**C**) List of the screening outputs.

The DPubChem tool allows the user to simply provide a bioassay accession number (AID) and the system automatically retrieves all relevant information for processing the HTS data of interest. The user can also provide a set of PubChem compound accession numbers (i.e., CID). Although the primary objective of the DPubChem tool is to derive QSAR models from PubChem data, the user may also input a list of simplified molecular-input line-entry system (SMILES)^24^ representing compounds of interest with their corresponding labels for target activity to build a model. Moreover, a list of AIDs may also be submitted for deriving an MLC model, where the correlation given by the common active compounds in different bioassays is exploited. In MLC models, each sample (a chemical compound, in our case) is assigned to multiple labels as opposed to just one label in binary or multi-class classification models^25,26^. This can be thought of as predicting properties of a chemical compound that are not mutually exclusive, such as, a chemical may be an activator in different bioassays (see Methods). MLC models have been applied to solve different problems in the bioscience domain and resulted in improved results compared to single label classification models^27–31^. Additionally, DPubChem implements a set of different feature selection methods to find an optimal subset of features and have demonstrated ability to reduce model complexity while, in some cases, enhances the prediction performance^32–35^. Since chemical compounds may be defined by thousands of different features (e.g., topological fingerprints, MACCS keys, among others), it is likely that not all of these features are relevant for the recognition of compound activities. Therefore, it is possible to derive simpler and possibly more robust QSR models by removing less relevant features from the initial set of features. Although feature selection methods need not improve the prediction performance for some recognition models, such as random forest (RF), the reduced subset of features still shortens training time and enables better model transparency^36^. Finally, DPubChem implements state-of-the-art solutions for addressing the class imbalance problem, which considerably increased the precision compared to other QSAR models for virtual screening (see Performance Evaluation subsection). Figure 1A shows the screenshot of the DPubChem interface for building a model.

For building QSAR models and predicting the biological activities of new compounds, DPubChem provides the following options: 1) a model is trained by using the parameters specified in the Model Building tab, 2) if a model has already been trained, the model is stored on our server and the jobID obtained from the Model Building page (Fig. 1A) may be directly used, 3) the user can select from a list of pre-trained models, and 4) the user can upload the files of a previously trained model using our system. The implemented options to upload model files or to input a jobID of an already trained model facilitate both the collaboration across different teams and the reproducibility of the results. Finally, DPubChem can build a visualization graph that measures chemical-chemical similarity, protein-protein similarity, bioassay-bioassay similarity, and assign activity screening scores for chemical-bioassay interactions^37^. Figure 1B shows a screenshot of the virtual screening page. Finally, DPubChem generates several statistical measures, an interactive network and a list of the screening outcome (Fig. 1C). Supplementary Material 1 shows the steps for building and testing a screening model in the tool.

### Performance evaluation

To evaluate the utility of the DPubChem tool, we first describe the results obtained by the state-of-the-art methods for addressing class imbalance and multi-label classification, followed by the performance obtained from 300 selected HTS assays.

In the context of virtual screening, a novel predicted interaction by the QSAR model may require further experimental validation. Therefore, it is crucial for the QSAR models to reduce the number of falsely predicted active compounds (false positives). In this case, precision is a meaningful statistical measure since a higher precision score indicates a lower number of false positives, or in other words, represents the proportion of all predictions denoted as active that are actually active. However, a very stringent QSAR model that only predicts few active compounds may achieve a high precision score while failing to identify most of the active compounds. For this, sensitivity is also important to consider and it represents the proportion of the active compounds correctly identified by the model. Therefore, we report the results in terms of the *F*_1_, which accounts for both precision and sensitivity. We also show the results in terms of the GMean of sensitivity and specificity to summarize prediction accuracy over both the true positive as well as the true negative rates. Both *F*_1_ and GMean incorporate false positives with different weight of importance. Specifically, *F*_1_ gives more preference to a lower number of false positives, while GMean reflects more the ability to identify the inactive class (i.e., true negatives). These two metrics are defined in Table 2.

**Table 2:** Selected statistical measures.

Statistical measure	Equation
*F*_*1*_	$\frac{2\times TP}{2\times TP+FP+FN}$
GMean	$\sqrt{\frac{TP}{TP+FN}\times \frac{TN}{TN+FP}}$

TP, TN, FN, and FP refer to true positives, true negatives, false negatives, and false positives, respectively.

**Figure 2:**
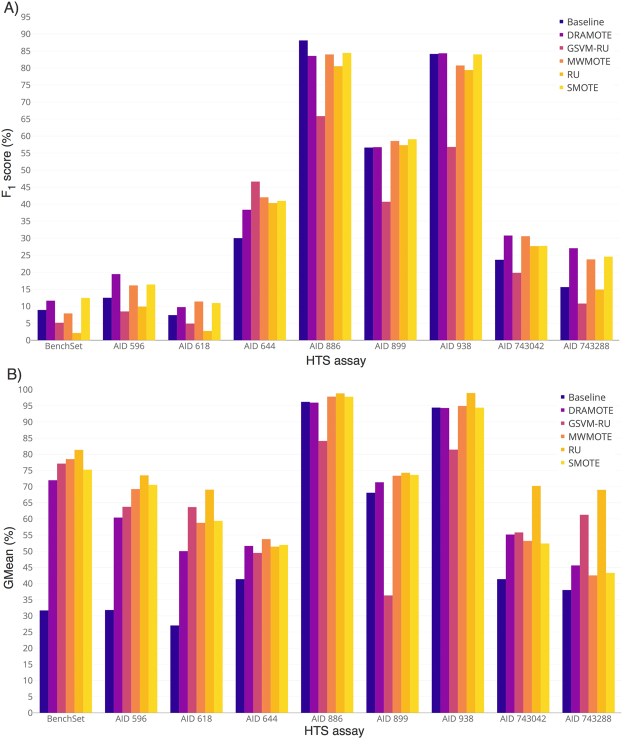
Average performance of the SVM-L, SVM-RBF, KNN, LDA, NBC, and RF from a 5-fold cross-validation of the implemented methods for reducing the effects of the class imbalance. (**A**) The results in terms of *F*_*1*_ for the 11 considered HTS assays. BenchSet refers to the pooled assays AIDs 773, 1006, and 1379 as described by Li *et al*.^70^. (**B**) The GMean results for the 11 HTS assays.

As mentioned above, reducing the number of false positive and false negative predictions is of critical importance for the virtual screening, but also a major challenge for the machine learning methods. As HTS assays usually contain a much higher number of inactive than active compounds (imbalanced labels), machine learning models tend to bias the majority class^38^. For this, we integrated the-state-of-the-art methods for solving the class imbalance problem (see Methods) into the DPubChem tool, namely, the Dragon Oversampling Technique (DRAMOTE)^39^, granular support vector machine for under-sampling (GSVM-RU)^40,41^, majority weighted minority over-sampling technique (MWMOTE)^42^, synthetic minority over-sampling technique (SMOTE)^43^, and the simple random under-sampling technique (RU). The methods were tested on 11 bioassays representing 487,557 active and inactive compounds. These datasets are characterized by different class imbalance ratios. Figure 2A,B show the average *F*_1_ and GMean from six classifiers, namely, support vector machines with linear kernel (SVM-L), support vector machine with radial basis function (SVM-RBF), K-nearest neighbor (KNN), linear discriminant analysis (LDA), naïve Bayes classifier (NBC), and RF using a 5-fold cross-validation, respectively. Supplementary Table S1 shows the number of compounds and the imbalance ratios of the HTS assays. From Fig. 2A, one observes that addressing the class imbalance enhanced the results showing an improvement of up to 55% compared to the baseline models (where the class imbalance is not addressed). The DRAMOTE and SMOTE, on average, achieved the best results except in assay AID 886. Figure 2B clearly demonstrates the effects of the unbalanced class labels when deriving machine learning models. In all the considered HTS assays, addressing the class imbalance considerably increased both the specificity and sensitivity and therefore, the GMean of the models. Notably, the BenchSet (AIDs 773, 1006, and 1379) showed the largest increase of the GMean by 247% compared to the baseline model. Although classification models are affected differently by the imbalance problem, an improvement of sensitivity (how well the model is able to recognize active compounds) was always observed when applying a class imbalance solution for the BenchSet dataset. Supplementary Table S2 shows the results obtained for each classification model on the BenchSet dataset. For instance, without addressing the class imbalance, we observe that RF was able to recognize all inactive compounds (100% specificity) but failed to identify the active compounds (only 4.06% sensitivity). Conversely, the sensitivity of the RF model when the class imbalance issue is addressed, increased to ~35–85% while conserving a high specificity (~85–100%).

However, it is not always necessary to address the class imbalance for certain HTS assays as they do not contain significant class imbalances. In these cases, it is possible to further improve the accuracy of the QSAR models by considering the correlation between assays given by the active compounds that are common in different HTS assays. As such, DPubChem implements the state-of-the-art technique Dragon Bayesian Active Learning (DRABAL)^44^, which consists of an MLC for modeling the correlations between several BioAssayDB assays (see Methods). The performance of DRABAL was tested on five assays (AIDs 1458, 485297, 485313, 588342, and 686978) representing 1,448,403 interactions with 7.7% hit rate indicating positive interactions. Using the 5-fold cross-validation, DRABAL achieved an *F*_1_ and GMean of 51.11% and 61.05%, respectively. These results represent a relative improvement of 14.27% and 9.91% for *F*_1_ and GMean, respectively, compared to the multi-label state-of-the-art methods.

Finally, to illustrate the applicability of DPubChem, we derived models for 300 datasets with different imbalance ratios and number of reported activities. The 300 selected datasets, reporting 116,751 activities, include bioassays with few compounds up to 11,000 (see Methods for the bioassay selection criteria). The imbalance ratio for these datasets ranged from 1:204 to 1:1. For each HTS assay, we used 80% of the data for training the model and the remaining 20% for testing with the default DPubChem options. The average *F*_1_ and GMean over the 300 assays are 76.68% and 76.53%, respectively. These results indicate a reasonable performance over the comprehensive set of HTS. Supplementary Table S3 shows the performance of the individual HTS assays. It is worth noting that the DPubChem tool provides a set of options for testing the QSAR model performance including cross-validation technique and holdout settings. The tool generates a report highlighting several performance measures, such as Cohen’s kappa coefficient, sensitivity, specificity, *F*_1_, and GMean, to help user judge the validity of the model and impact of potential noise.

### Case study analysis

**Figure 3:**
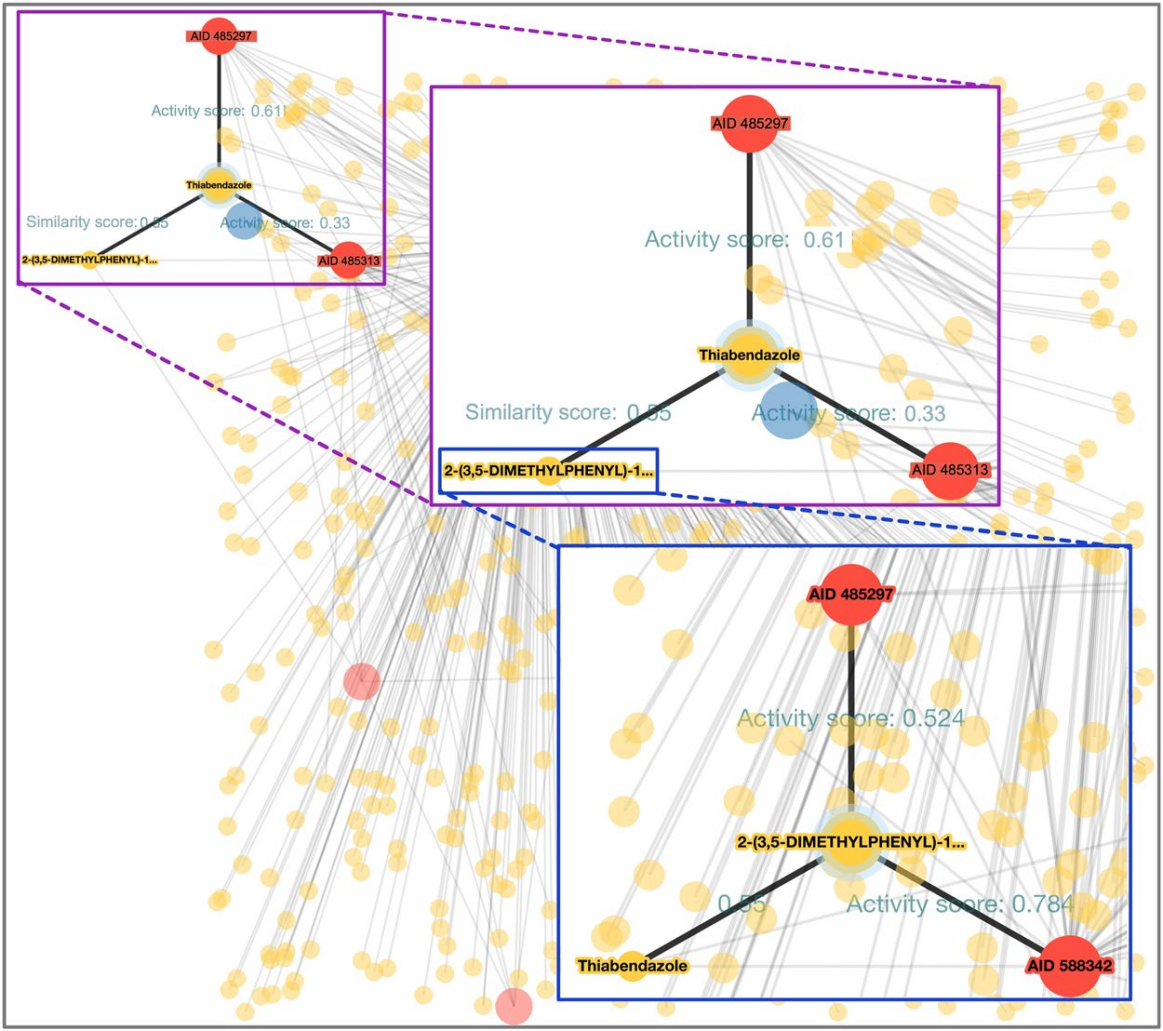
DPubChem interaction network obtained from applying DRABAL on the five selected HTS assays (AIDs 1458, 485313, 485297, 588342, and 686978). Thiabendazole (DB00730) is the top common prediction for assays AIDs 485297 and 485313. In the graph, red, yellow, and blue colored nodes indicate HTS assays from PubChem database, chemicals, and the biological target entities like protein targets, respectively. The interaction network is accessible at www.cbrc.kaust.edu.sa/dpubchem/DraPubChemGraph/drapubchemgraph.html and is obtained by running DRABAL MLC method on the selected HTS assays.

In order to show the utility of DPubChem and the interaction networks, we screened the FDA approved drugs in five HTS assays, namely, AID 1458, AID 485313, AID 485297, AID 588342, and AID 686978. Based on the interaction network produced by DPubChem, we generated Fig. 3 aiming to highlight the interactions of relevance for the case study. Figure 3 shows the interactions between HTS assays and compounds with their predicted activity scores (see Methods) that enable us to suggest potential drug-target interactions of interest. Specifically, we focused on the results for bioassays AID 485313 (target protein being: Ras-related protein Rab-9A) and AID 485297 (target protein being: Niemann-Pick C1 protein precursor) as both of these target proteins that could potentially serve as targets of drugs for Niemann-Pick type C (NPC) disease. The prediction of interactions by DRABAL^44^ component of DPubChem and resultant interaction network showed that Thiabendazole (DrugBank Database ID: DB00730) is the strongest common activator of HTS assays AID 485313 and AID 485297. Since overexpression of both Rab-A9 and NPC1 proteins have been shown to reduce the symptoms of the NPC disease^45,46^, we hypothesized that the common predicted activator (Thiabendazole) may inhibit the progression of the NPC disease. Additionally, Thiabendazole is an aryl hydrocarbon receptor ligand that has been shown to reduce levels of cathepsin D^47^, a protein which overexpression has been implicated in some of the symptoms of the NPC disease, apoptosis^48^, and liver fibrosis^49^. Finally, we note that both Thiabendazole (predicted activator) and Benzoic Acid (approved drug DB03793 to target Rab-9A protein) belong to the same Benzenoid superclass. Although Thiabendazole is deemed slightly toxic and is actively used as a pesticide, pharmacokinetics studies of Thiabendazole report that ∼87% of the oral dose in humans excretes within 24 hours and similarly in animals^50^. Therefore, Thiabendazole may be beneficial if administered conservatively^44^.

Although this case study focuses on Thiabendazole for the potential inhibition of NPC disease, interaction networks are a powerful tool for the identification of other chemical compounds common to multiple bioassays. Moreover, while QSAR models predict the activity of a candidate chemical compound for a specific bioassay, the similarities between these compounds provide another layer of information. Inspired by the underlying idea that similar compounds are likely to interact with similar proteins, these networks provide a graphical representation that may facilitate the identification of similar compounds to those known to be active within a given bioassay. Additionally, based on Fig. 3 (bottom right), we may hypothesize that compound 2-(3,5-Dimethylphenyl)-1,3-Benzoxazole may also have an effect in the NPC disease as it is similar to Thiabendazole (similarity score of 0.55) and a has a predicted activity in AID 485297 (activity score of 0.524). Clearly, a compound with a higher similarity score to Thiabendazole would indicate a better candidate.

## Discussion

With the vast amount of data from public repositories that provide access to biological activity information from HTS experiments (e.g., BioAssayDB), there is an opportunity to develop categorical models to predict the biological activities of millions of chemical compounds that remain untested. Although several QSAR models have been proposed, they remain limited in many aspects. Some of these tools lack flexibility or impose a set of different steps for data processing that are complicated and time-consuming. Moreover, these tools have a low precision of predictions, i.e., high false positive rates. Consequently, we developed DPubChem, a tool that enables sophisticated virtual screening strategies based on state-of-the-art machine learning methods for feature selection, class imbalance, and MLC. The DPubChem tool focuses on the simplicity of the interface without compromising the flexibility and in the precision of the QSAR models to reduce false positive predictions. Because DPubChem uses a single easy-to-use workflow that supports a different set of models and options, it is straightforward to generate different models for the same screening task. Each of these models provides the performance statistics, predictions ranking, and the graph visualization, which allows the user to easily select the best performing model. The notable ease-of-use of this tool is essential for users. The prediction results from DPubChem may be further examined by published results in the literature and other computational techniques like 3D docking simulations. Although docking simulations are prone to false positives, they can indirectly support the top predictions from our data-driven approach^39^, especially, if an experimental validation (i.e., *in vitro* and *in vivo* based) is prohibitive to run. Currently, DPubChem does not explicitly allow for such docking simulation types of verifications. Nevertheless, it provides visualization graphs with reference links that can be used to get more background information. We believe that DPubChem will contribute to the progress of bioinformatics and biomedical research.

## Methods

**Figure 4:**
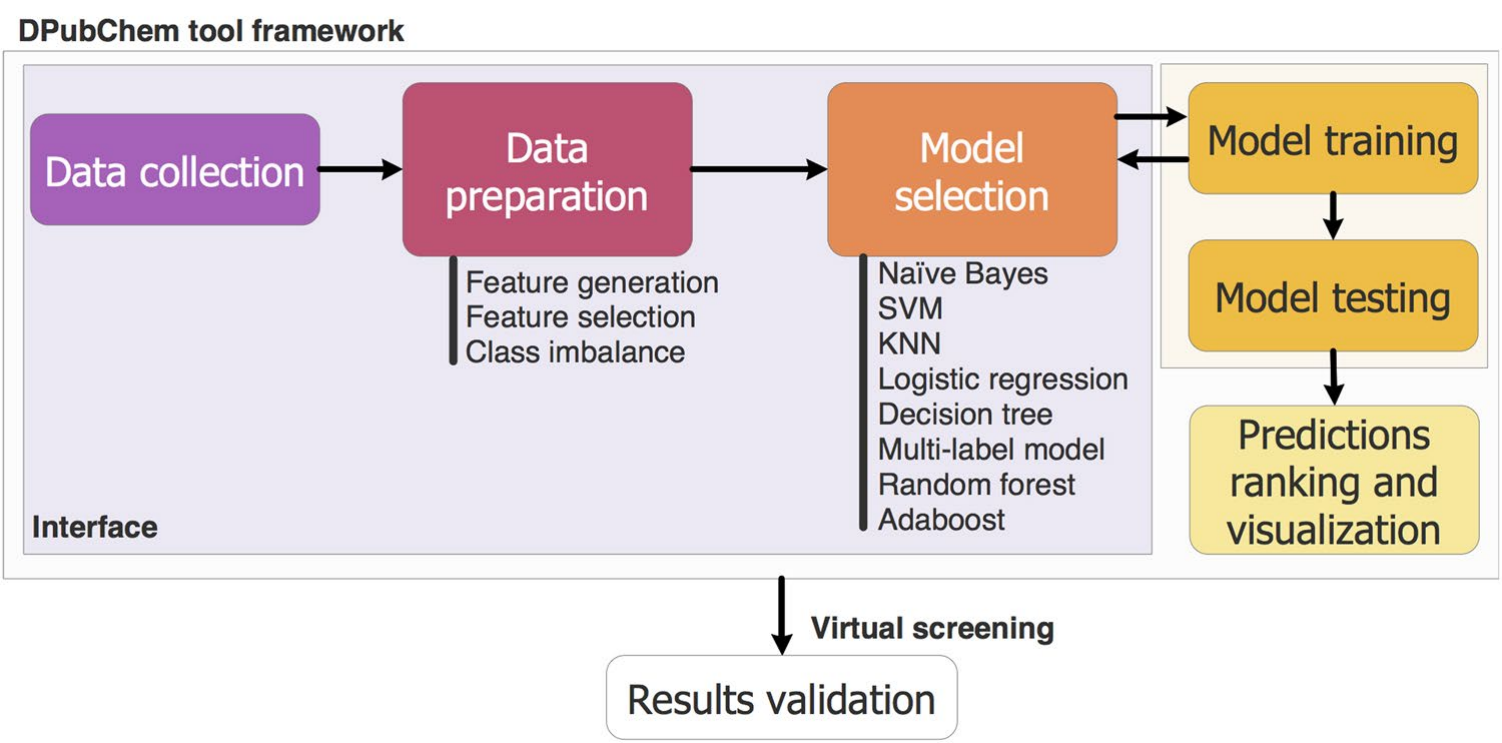
DPubChem framework for virtual screening. Different colors indicate the four modules that represent the core architectural design of DPubChem.

The framework in Fig. 4 depicts the core architectural design of DPubChem. In particular, we propose four modules to enable virtual screening of HTS assays using machine learning methods. The first module is the data collection, in where the user may input 1) a BioAssay accession number (AID), 2) a list of AIDs for an MLC model, 3) a list of PubChem compound accession numbers (CID), and 4) a list of SMILES records with their corresponding labels. The next is the data preparation module, where the chemical compounds are described by a set of features (feature generation), which can be further reduced by a feature selection method. The third module is for the model selection, where different classifiers may be selected. Finally, the QSAR models for virtual screening are derived, and the predictions are ranked and visualized in an interaction network. The following subsections describe these modules in more details.

### Data collection

The datasets retrieved by DPubChem are based on the PubChem BioAssay protocol, where datasets represent HTS assays that can be referenced by a unique AID identifier. We considered bioassays that report experimental activity results for a set of chemical compounds over a specific biological target (e.g., a protein). Therefore, a bioassay dataset contains a list of chemical compounds to which we assign labels, where label ‘1’ indicates that the compound appears active in the assay, while ‘2’ relates to inactive compounds. The probe designation was considered as active (label of 2) as it indicates that the activity of the test result has been tested and confirmed through multiple rounds of experimental inquiry^1^. Inconclusive or unspecified activity types are ignored. The criteria for selecting the 300 considered datasets are: 1) since sufficient information about both classes is needed to build meaningful recognition models only confirmatory assays with more than five samples for both active and inactive classes were considered, 2) to have a reasonable processing time, we selected assays containing at most 11,000 reported active compounds, and 3) the 300 datasets were randomly selected. Although some of these datasets contain a relatively small number of compounds, models derived from these datasets allow for HTS.

### Data preparation and feature selection

In order the build QSAR models, chemicals are encoded into a set of features. The generation and selection of a representative subset of features are critical for developing accurate QSAR models^33^. DPubChem implements different types of chemical features, namely, 1) the PubChem fingerprints^51^ (881 features from ftp://ftp.ncbi.nlm.nih.gov/pubchem/specifications/pubchem_fingerprints.txt), 2) MACCS keys fingerprints from the toolkit OpenBabel^52^(166 features), 3) the topological fingerprints from the toolkit RDKit^53^ (1,024 features), 4) chemical descriptors, i.e., the number of H-acceptors and donors, molecular weight, and Log-P, among others (166 features), 5) a standard set of features, representing a combination of all the previous types of features, and 6) a recommended set of chemical features, DWFS. The DWFS set of features is the result of an extensive analysis and feature selection process, which resulted in an optimized subset of 1,064 chemical features that enabled superior predictor performance^39,44,54^. However, there is no guarantee that this optimized set of features will be optimal for all cases.

Feature selection is crucial for removing irrelevant or redundant features that do not contribute to the performance of the QSAR models. This results in simpler and possibly more accurate models. For this, DPubChem provides efficient solutions to select the most relevant features. The current implementation incorporates the following state-of-the-art methods for feature selection from the MATLAB Feature Selection Tool (FEAST)^55^: minimum redundancy maximum relevance (mRMR)^34^, joint mutual information (JMI)^34^, conditional mutual information maximization (CMIM)^56^, and RELIEF^57^. Moreover, DPubChem also includes the simpler algorithms for feature selection based on the correlation of features and the standard deviation.

### Classification model selection

Seven widely used classifiers are available in DPubChem as a basis for building prediction models for PubChem assays. These include SVM-L and SVM-RBF^58^, K-NN^59^, decision trees^60^, NBC^61^, LDA, and ensemble classifiers RF^62^ and Adaboost^63^ from the Scikit learn machine learning package^64^. There are several justifications for the selection of the implemented classifications models. Although deep neural networks have achieved superior performance over shallow models in some applications^38,65^, the training and tuning of such complex models are computationally-demanding, especially for large datasets as are many in the PubChem assays. Therefore, we have included less computationally-demanding models. Additionally, an extensive empirical study^66^ tested the performance of different classification models over the UCI machine-learning repository database^67^ and showed that SVM and ensemble models (random forest and Adaboost) were among the top-ranked models.

#### Multi-label classification model

**Figure 5:**
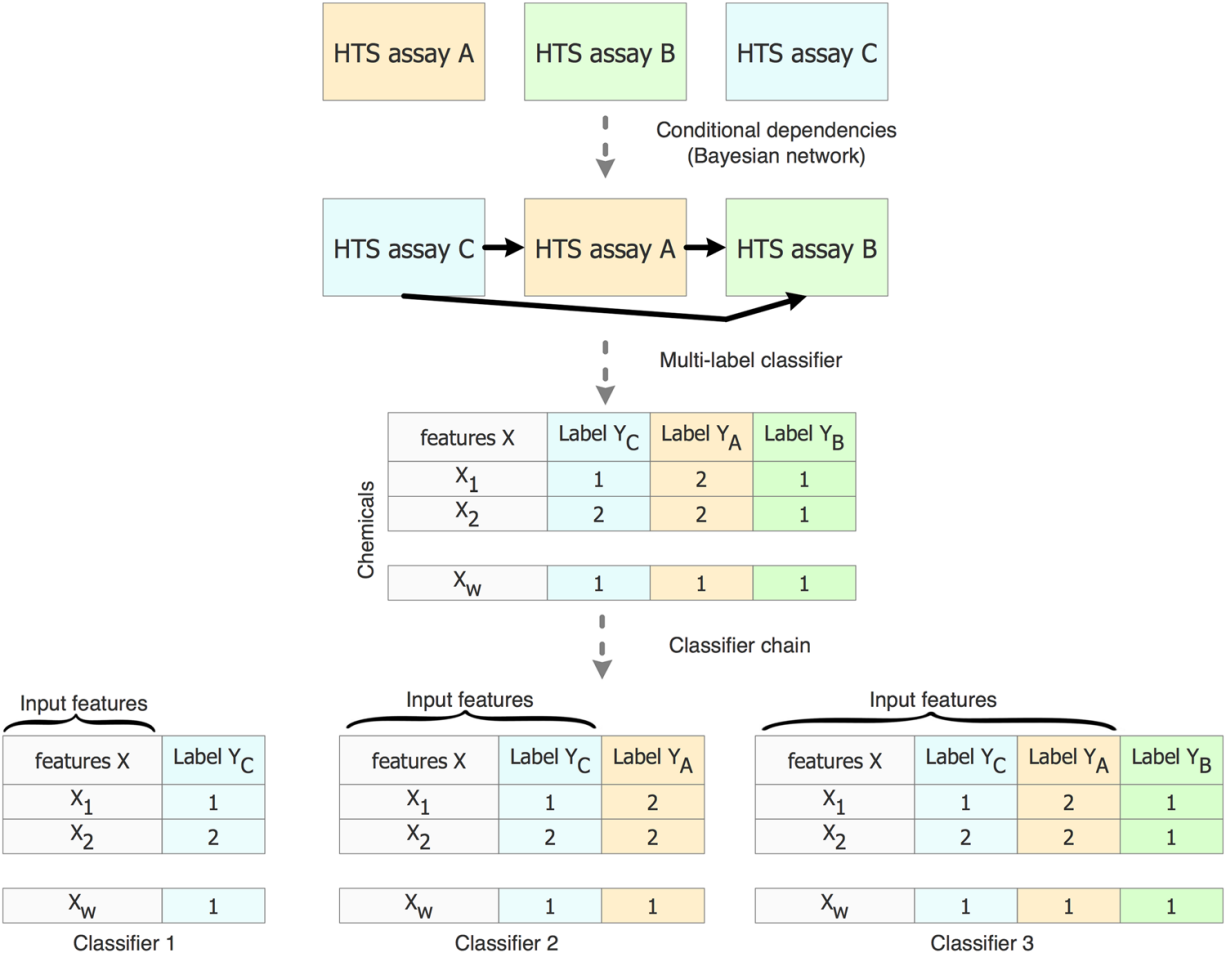
Multi-label classification model using classification chain transformation.

Since various HTS assays in BioAssayDB are correlated by sharing some portion of the same set of active compounds, we have included DRABAL^44^, an MLC technique for modeling correlations between several BioAssayDB assays to enhance prediction performance. DRABAL uses a problem transformation method to derive MLC models. This method transforms the MLC problem into a chain of *n* single label classifiers, where *n* denotes the number of labels assigned to a chemical compound. For this, the first classifier is derived by using the input data to fit one label, and then each of the next *n* classifiers is trained on the original input data and the labels of the previous classifiers (i.e., target labels are concatenated to the original set of features). For example, the last classifier in the chain, classifier *n*, would be derived by using the original input data and the *n-*1 target labels. This implies that the order of the labels has to be specified. For this, DRABAL uses a Bayesian network to learn the correlation of the target labels of the assays^44^. Figure 5 shows an example of an MLC model for tree HTS assays.

#### Class imbalance problem in HTS assays

Given the nature of HTS assays, which are often characterized by a small number of active chemical compounds obtained after screening a big compound set library, we implemented several state-of-the-art solutions in DPubChem to address the class imbalance problem. In several cases, addressing the class imbalance has shown to overcome possible bias to the majority class and achieved considerably better results than without any data preprocessing. Depending on the imbalance ratio and size of active compounds, different solutions can lead to different QSAR model performance. DPubChem offers seven combinations that can result in a different effect on both sensitivity and precision of virtual screening. The current options include the following approaches: RU^39^, SMOTE^43^, MWMOTE^42^, and the precision-aware method called DRAMOTE^39^.

### Interaction networks

Given a set of chemical compounds $C=\{{c}_{1},{c}_{2},\ldots ,{c}_{n}\}$, a set of proteins $P=\{{p}_{1},{p}_{2},\ldots ,{p}_{n}\}$, and a set of assays $A=\{{a}_{1},{a}_{2},\ldots ,{a}_{n}\}$, the interaction network can be generated to represents nodes from *C*, *P*, and *A* and their links. Chemical-chemical links (weighted edges between ${c}_{1},{c}_{2}\in C$) and protein-protein links (weighted edges between ${p}_{1},{p}_{2}\in P$) represent the similarity scores between the two nodes and are computed by using the SIMComp^68^ and Smith-Waterman^69^ methods, respectively. The chemical compound-bioassay interaction links (weighted edges between $c\in C,a\in A$) denote the predicted probability of a compound to be active by the QSAR model. The similarity scores and the probability of interactions are values within the range of [0, 1].

## Electronic supplementary material

Supplementary Material
