# Supplementary material for: DPubChem: a web tool for QSAR modeling and high-throughput virtual screening
Source: Sci Rep. 2018 Jun 14;8:9110. doi: 10.1038/s41598-018-27495-x (PMC6002400; doi:10.1038/s41598-018-27495-x)
Supplement: Supplementary file 1 — Supplementary Material [file 41598_2018_27495_MOESM1_ESM.pdf]

DPubChem: a web tool for QSAR modeling and high-throughput virtual screening

Othman Soufan[1], Wail Ba-alawi[2,3], Arturo Magana-Mora[4], Magbubah Essack[5] and Vladimir B. Bajic[5,*]

[1]Institute of Parasitology, McGill University, Montreal, QC, H9X 3V9, Canada.

[2]Princess Margaret Cancer Centre, University Health Network, Toronto, ON, M5G 1L7, Canada.

[3]Department of Medical Biophysics, University of Toronto, Toronto, ON, M5G 1L7, Canada.

[4]Computational Bio Big-Data Open Innovation Laboratory (CBBD-OIL), National Institute of Advanced Industrial Science and Technology (AIST), Tokyo, 135-0064, Japan.

[5]Computational Bioscience Research Center, King Abdullah University of Science and Technology (KAUST), Thuwal, 23955-6900, Saudi Arabia.

*vladimir.bajic@kaust.edu.sa

Supplementary Tables

Supplementary Table S1. Performance of the implemented methods for solving the class imbalance problem.

Assay	Minority class size	Majority class size	Ratio	Method	F1 score	Gmean
BenchSet	478	184,154	1:377	RU	2.11	81.36
				GSVM-RU	5.13	77.12
				SMOTE	12.44	75.24
				MWMOTE	7.87	78.5
				DRAMOTE	11.62	71.97
				Baseline	8.89	31.68
AID 596	1,391	66,726	1:48	RU	9.89	73.48
				GSVM-RU	8.46	63.74
				SMOTE	16.38	70.54
				MWMOTE	16.11	69.24
				DRAMOTE	19.43	60.42
				Baseline	12.47	31.81
AID 618	537	86,197	1:160	RU	2.7	69.06
				GSVM-RU	4.89	63.66
				SMOTE	10.93	59.4
				MWMOTE	11.38	58.78
				DRAMOTE	9.73	50.03
				Baseline	7.38	27.03
AID 644	67	139	1:2	RU	40.32	51.43
				GSVM-RU	46.62	49.48
				SMOTE	40.95	51.96

				MWMOTE	41.99	53.75
				DRAMOTE	38.34	51.64
				Baseline	30	41.37
AID 886	2,463	64,616	1:26	RU	80.52	98.86
				GSVM-RU	65.86	84.12
				SMOTE	84.43	97.81
				MWMOTE	83.98	97.84
				DRAMOTE	83.55	95.99
				Baseline	88.1	96.22
AID 899	1,901	6,443	1:3	RU	57.33	74.28
				GSVM-RU	40.69	36.32
				SMOTE	59.07	73.62
				MWMOTE	58.55	73.36
				DRAMOTE	56.73	71.34
				Baseline	56.62	68.1
AID 938	1,794	60,806	1:34	RU	79.4	98.97
				GSVM-RU	56.79	81.44
				SMOTE	84	94.43
				MWMOTE	80.74	94.95
				DRAMOTE	84.32	94.33
				Baseline	84.13	94.45
AID 743042	674	6,939	1:10	RU	27.66	70.21
				GSVM-RU	19.81	55.82
				SMOTE	27.71	52.4
				MWMOTE	30.56	53.19
				DRAMOTE	30.76	55.17
				Baseline	23.63	41.37
AID 743288	95	2,128	1:22	RU	14.89	69
				GSVM-RU	10.78	61.28
				SMOTE	24.56	43.3
				MWMOTE	23.75	42.52
				DRAMOTE	27.03	45.61
				Baseline	15.62	38

Supplementary Table S2. 5-fold cross-validation performance on BenchSet for the implemented class imbalance solutions for each classifier separately.

Classifier	Algorithm	Sensitivity	Specificity	Precision	GMean	F_1 score	ROC AUC
SVM-L	Baseline	0.17%	**100.00%**	0.00%	1.87%	0.00%	11.22%
	SMOTE	64.30%	96.42%	**4.60%**	78.72%	8.57%	93.63%
	MWMOTE	65.46%	93.95%	2.82%	78.35%	5.41%	8.35%
	GSVM-RU	62.02%	97.32%	5.97%	77.67%	**10.86%**	**94.05%**
	RU	**81.85%**	80.44%	1.11%	**81.10%**	2.18%	89.18%
	DRAMOTE	57.40%	96.70%	4.43%	74.47%	8.22%	92.76%
SVM-RBF	Baseline	0.00%	**100.00%**	0.00%	0.00%	0.00%	9.37%
	SMOTE	57.63%	98.39%	8.73%	75.25%	15.13%	**94.43%**
	MWMOTE	75.32%	94.54%	3.55%	**84.36%**	6.77%	5.15%
	GSVM-RU	63.99%	89.85%	1.70%	75.69%	3.30%	86.46%
	RU	**85.27%**	77.59%	1.01%	81.32%	2.00%	90.87%
	DRAMOTE	56.61%	98.67%	**10.23%**	74.71%	**17.28%**	94.38%
KNN	Baseline	14.79%	**99.95%**	**45.96%**	38.37%	**22.20%**	70.04%
	SMOTE	74.44%	86.88%	1.49%	80.39%	2.91%	85.24%
	MWMOTE	71.41%	87.47%	1.49%	79.02%	2.93%	83.78%
	GSVM-RU	71.95%	72.53%	0.70%	72.14%	1.38%	79.80%
	RU	**86.49%**	69.50%	0.75%	77.52%	1.49%	84.63%
	DRAMOTE	78.16%	83.24%	1.22%	**80.62%**	2.41%	**85.39%**
LDA	Baseline	27.21%	**99.67%**	**18.11%**	51.98%	**21.69%**	92.74%
	SMOTE	67.08%	95.79%	4.06%	80.12%	7.65%	93.73%
	MWMOTE	80.38%	90.83%	2.29%	**85.44%**	4.45%	**93.97%**
	GSVM-RU	67.42%	95.61%	3.97%	80.24%	7.49%	93.18%
	RU	**89.60%**	81.37%	1.27%	85.37%	2.50%	92.85%
	DRAMOTE	68.65%	95.55%	3.95%	80.97%	7.46%	93.41%
NBC	Baseline	81.90%	75.17%	0.87%	**78.45%**	1.73%	85.53%
	SMOTE	78.03%	77.48%	0.92%	77.74%	1.82%	83.95%
	MWMOTE	79.34%	74.67%	0.83%	76.97%	1.64%	82.87%
	GSVM-RU	78.64%	76.10%	0.87%	77.36%	1.72%	**85.85%**
	RU	**85.81%**	70.78%	0.78%	77.92%	1.54%	84.46%
	DRAMOTE	65.61%	**83.89%**	**1.15%**	73.87%	**2.26%**	82.12%
RF	Baseline	4.06%	**100.00%**	**93.33%**	19.41%	7.70%	85.07%
	SMOTE	35.26%	99.88%	42.84%	59.24%	**38.55%**	**93.77%**
	MWMOTE	45.02%	99.46%	18.43%	66.86%	26.04%	94.18%
	GSVM-RU	67.18%	94.48%	3.17%	79.60%	6.04%	89.10%
	RU	**85.02%**	84.87%	1.49%	**84.93%**	2.93%	93.16%
	DRAMOTE	22.44%	99.96%	59.10%	47.16%	32.07%	92.36%

Supplementary Table S3. DPubChem performance over 300 datasets. Each model was derived by using 80% of the data and tested on the remaining 20%.

BioAssay ID	Minority class	Majority class	Imbalance Ratio (%)	Precision (%)	GMean (%)	F_1 score (%)
410	4112	3672	89.30	84.86	82.13	82.99
450	110	9725	1.13	88.97	86.37	81.20
451	38	7755	0.49	97.78	93.60	92.42
466	266	242	90.98	58.49	58.09	60.74
550	136	184	73.91	66.90	68.63	62.93
569	29	55	52.73	87.00	68.16	64.63
621	80	95	84.21	73.05	82.58	76.66
649	14	15	93.33	80.00	65.32	64.00
669	8	21	38.10	100.00	77.46	75.00
671	14	15	93.33	80.00	70.83	67.47
676	23	23	100.00	85.83	74.95	77.69
702	34	24	70.59	70.86	68.08	74.91
726	27	24	88.89	76.67	73.33	68.15
728	48	34	70.83	96.00	88.06	91.24
733	194	196	98.98	67.76	65.83	64.83
735	15	27	55.56	73.33	69.28	61.75
755	25	19	76.00	74.22	68.19	81.35
776	21	23	91.30	82.00	74.34	75.33
810	110	100	90.91	69.00	68.46	71.46
820	37	35	94.59	83.93	74.51	76.90
825	48	50	96.00	69.67	62.96	61.76
831	34	95	35.79	100.00	76.04	73.27
846	91	198	45.96	89.47	80.67	77.11
851	54	27	50.00	81.89	82.01	88.87
852	146	495	29.49	95.11	78.00	74.69
854	24	39	61.54	76.67	79.91	72.45
856	55	26	47.27	83.00	83.75	88.50
884	3410	7061	48.29	86.10	84.25	80.19
891	1550	6334	24.47	81.43	71.35	63.83
899	1811	6440	28.12	81.42	71.18	63.72
911	24	22	91.67	58.67	63.31	64.57
914	220	7610	2.89	76.42	73.62	63.61
943	154	600	25.67	91.07	73.87	69.24

1000	36	21	58.33	100.00	98.74	98.73
1011	15	12	80.00	75.00	69.40	79.69
1028	28	48	58.33	100.00	94.06	94.89
1052	45	67	67.16	85.53	78.34	80.25
1054	64	69	92.75	82.12	78.44	82.61
1055	61	128	47.66	78.06	75.35	72.67
1056	213	255	83.53	78.52	76.91	76.05
1059	57	55	96.49	84.07	84.79	85.85
1070	66	75	88.00	76.64	81.44	79.03
1072	31	21	67.74	65.83	75.30	74.38
1077	145	96	66.21	81.97	78.70	85.66
1083	29	132	21.97	79.17	89.91	81.87
1193	21	32	65.63	58.10	64.57	61.50
1215	223	294	75.85	95.18	80.79	78.69
1231	46	24	52.17	76.19	78.31	81.81
1392	15	31	48.39	80.00	74.74	71.93
1398	60	62	96.77	57.66	59.19	53.89
1418	509	667	76.31	76.29	77.69	73.80
1491	63	114	55.26	93.06	75.59	72.78
1512	93	169	55.03	88.27	81.64	80.50
1518	17	14	82.35	74.44	69.81	79.37
1535	123	70	56.91	83.57	75.62	85.55
1540	116	8	6.90	95.16	70.42	97.12
1558	44	52	84.62	67.51	69.32	69.98
1573	27	20	74.07	69.60	71.32	76.44
1641	92	118	77.97	70.04	63.92	60.10
1655	48	127	37.80	73.16	70.75	63.11
1666	27	42	64.29	65.00	68.20	59.97
1686	22	24	91.67	61.67	71.47	65.57
1689	9	31	29.03	80.00	80.28	72.73
1751	90	23	25.56	98.09	91.05	98.59
1770	332	794	41.81	89.18	91.83	88.37
1771	356	747	47.66	91.35	93.01	90.50
1795	178	995	17.89	84.41	82.00	75.78
1821	48	52	92.31	59.71	59.65	57.93
1869	50	135	37.04	95.56	75.92	72.29
1872	27	158	17.09	100.00	70.95	66.96

1884	54	131	41.22	93.33	71.64	67.01
1888	53	132	40.15	100.00	78.22	75.92
1890	44	57	77.19	68.06	64.27	61.60
1897	93	92	98.92	64.27	64.43	61.53
1957	35	40	87.50	84.17	79.77	80.67
1958	26	25	96.15	75.95	70.50	76.86
1960	696	635	91.24	75.05	73.42	76.09
2001	263	282	93.26	61.83	60.83	58.73
2018	602	631	95.40	73.66	73.27	71.18
2019	32	60	53.33	67.70	83.68	75.39
2020	24	68	35.29	83.33	87.99	85.76
2027	28	23	82.14	85.33	83.22	81.01
2051	30	21	70.00	75.81	79.16	77.23
2080	23	19	82.61	73.33	64.77	72.83
2091	133	234	56.84	80.37	70.73	65.08
2110	54	41	75.93	92.60	90.21	90.41
2111	54	39	72.22	87.83	86.68	88.85
2113	53	331	16.01	86.77	87.42	82.06
2115	15	303	4.95	85.00	86.31	79.69
2121	114	123	92.68	76.72	75.77	73.13
2136	44	166	26.51	75.62	71.86	63.00
2158	286	229	80.07	89.05	86.69	87.01
2161	158	235	67.23	79.64	77.12	72.42
2162	25	52	48.08	100.00	88.10	87.40
2230	92	99	92.93	82.95	79.15	81.86
2263	24	24	100.00	100.00	98.56	100.00
2265	17	32	53.13	86.67	89.13	84.97
2284	15	15	100.00	70.00	72.16	70.82
2295	16	16	100.00	85.00	74.98	84.67
2296	14	18	77.78	53.33	67.00	59.39
2333	188	96	51.06	79.64	71.05	86.55
2334	45	86	52.33	66.39	70.58	62.70
2337	157	128	81.53	72.14	74.38	78.42
2359	21	41	51.22	81.00	81.25	78.06
2366	10	16	62.50	51.67	66.33	60.62
2400	157	182	86.26	63.22	60.07	56.35
2444	17	12	70.59	83.33	71.78	85.76

2479	21	14	66.67	86.00	93.99	92.47
2480	36	24	66.67	86.00	73.30	78.17
2483	8	12	66.67	90.00	75.50	72.00
2490	39	27	69.23	85.43	81.93	85.02
2497	8	8	100.00	60.00	73.48	72.00
2500	14	10	71.43	88.33	87.18	91.55
2515	10	9	90.00	90.00	81.24	90.00
2530	46	192	23.96	93.50	84.21	81.18
2533	81	115	70.43	92.57	93.32	93.60
2559	58	67	86.57	84.92	82.30	82.30
2561	37	143	25.87	87.79	89.68	85.92
2567	59	67	88.06	86.95	77.47	77.08
2568	70	78	89.74	85.11	78.42	74.59
2576	24	96	25.00	90.00	80.00	77.22
2588	56	47	83.93	80.61	82.27	81.56
2590	156	59	37.82	86.07	79.32	91.25
2595	24	14	58.33	82.50	72.87	85.86
2613	26	42	61.90	74.44	78.83	73.29
2614	81	28	34.57	91.43	75.53	92.97
2654	31	55	56.36	55.00	66.39	57.74
2669	77	58	75.32	78.02	71.60	73.67
2671	43	33	76.74	94.29	91.29	91.24
2673	25	89	28.09	62.95	78.55	66.14
2678	31	24	77.42	72.67	65.14	70.69
2684	44	39	88.64	75.24	73.44	77.94
2708	168	321	52.34	73.43	68.11	59.56
2733	35	29	82.86	63.80	67.07	70.75
2753	114	127	89.76	62.56	59.36	56.04
2755	37	56	66.07	83.61	75.06	70.80
2758	32	93	34.41	85.00	75.02	69.09
2763	97	96	98.97	62.02	57.23	56.06
2784	214	171	79.91	63.82	61.45	65.45
2799	9	30	30.00	53.33	69.23	54.15
2808	23	82	28.05	92.00	72.21	67.71
434957	13	24	54.17	88.33	87.66	88.33
434981	32	26	81.25	77.78	75.14	80.35
435011	43	43	100.00	67.24	64.62	66.15

435026	407	606	67.16	84.05	73.60	69.09
449726	48	34	70.83	76.88	67.55	80.33
449727	56	27	48.21	79.05	68.31	85.00
449736	62	28	45.16	82.47	80.18	84.37
449737	35	31	88.57	77.33	64.14	76.21
449766	17	40	42.50	76.67	77.44	70.55
463105	18	16	88.89	60.00	59.63	63.16
463184	20	9	45.00	100.00	97.98	97.96
463185	32	24	75.00	95.00	87.99	86.91
463214	204	181	88.73	74.90	73.41	77.03
463217	20	10	50.00	88.33	78.82	82.66
463250	54	31	57.41	74.36	63.94	77.04
485277	10	57	17.54	90.00	94.23	90.00
485285	42	25	59.52	77.78	77.39	81.48
485337	16	14	87.50	92.00	84.04	88.23
485351	34	48	70.83	89.17	86.88	84.76
485374	66	11	16.67	90.00	77.78	92.21
485391	62	20	32.26	85.70	82.73	89.35
488788	278	275	98.92	61.14	63.36	61.57
488795	23	89	25.84	82.00	71.06	64.39
488808	42	70	60.00	69.61	74.22	67.44
488810	40	112	35.71	93.00	85.57	84.06
488819	23	18	78.26	85.33	81.30	83.95
488821	30	73	41.10	76.07	82.83	76.10
488828	50	36	72.00	91.36	90.23	90.40
488833	20	14	70.00	80.00	64.08	77.24
488836	18	23	78.26	74.00	69.48	77.54
488838	41	40	97.56	82.50	71.91	76.70
488854	34	14	41.18	79.67	69.24	85.72
488914	14	10	71.43	76.67	70.00	79.09
488920	15	12	80.00	80.00	90.74	86.86
488925	30	14	46.67	90.17	76.56	90.17
489010	76	60	78.95	80.03	82.70	87.79
489012	139	129	92.81	65.34	71.82	71.86
489036	45	56	80.36	82.81	84.57	84.56
489037	89	40	44.94	83.29	73.43	88.38
489038	21	41	51.22	92.67	94.32	93.00

489039	30	30	100.00	93.81	91.64	90.86
489043	175	109	62.29	75.53	69.76	82.97
492948	201	76	37.81	84.64	72.14	90.40
492949	118	89	75.42	77.50	78.65	83.92
492987	16	29	55.17	78.00	69.72	66.75
492994	23	22	95.65	83.33	85.63	83.17
493041	24	8	33.33	85.33	73.37	89.91
493073	809	432	53.40	80.80	74.92	86.08
493094	13	26	50.00	70.00	88.19	76.09
493102	809	432	53.40	81.31	75.15	86.54
493112	27	30	90.00	82.50	79.07	79.78
493113	12	17	70.59	55.00	72.11	60.27
493126	34	23	67.65	80.14	71.65	78.94
493132	44	13	29.55	85.56	80.05	90.53
493135	12	56	21.43	88.33	89.29	85.76
493141	14	64	21.88	90.00	81.84	78.75
493142	26	52	50.00	87.50	89.29	85.37
493177	809	432	53.40	80.93	75.28	86.32
493205	45	61	73.77	84.20	78.74	77.00
493206	117	7	5.98	98.28	70.71	99.13
493231	163	233	69.96	71.92	77.55	73.95
504428	24	40	60.00	78.95	86.12	84.98
504488	117	149	78.52	77.09	76.21	73.16
504492	110	156	70.51	61.01	67.47	59.03
504497	141	125	88.65	72.89	71.82	72.22
504537	21	11	52.38	85.33	69.28	90.35
504559	12	16	75.00	63.33	64.16	63.33
504687	103	25	24.27	91.72	82.16	92.73
504694	12	16	75.00	63.33	64.16	63.33
504730	44	60	73.33	65.81	67.04	59.28
504763	89	129	68.99	75.44	73.38	67.02
504792	39	547	7.13	76.00	74.72	64.77
540269	275	311	88.42	71.17	65.65	60.44
540322	66	40	60.61	82.26	80.88	86.89
540353	91	56	61.54	71.14	65.91	76.39
540359	79	88	89.77	75.75	73.08	70.99
540371	15	16	93.75	65.00	72.67	70.77

588341	140	95	67.86	75.48	69.41	78.72
588384	12	15	80.00	80.00	93.99	88.89
588385	10	17	58.82	60.00	84.68	73.55
588387	11	16	68.75	60.00	90.11	73.55
588388	11	16	68.75	60.00	90.11	73.55
588390	9	18	50.00	50.00	69.54	53.13
588394	12	15	80.00	80.00	93.99	88.89
588415	20	48	41.67	90.00	81.00	78.54
588439	32	199	16.08	67.67	70.00	58.36
588463	13	1134	1.15	90.00	81.62	76.60
588495	12	108	11.11	56.00	78.19	59.44
588503	9	38	23.68	71.67	80.92	72.49
588515	19	1858	1.02	95.00	87.54	84.85
588525	44	27	61.36	84.29	80.63	89.12
588552	28	30	93.33	58.57	59.72	57.75
588566	62	26	41.94	80.75	68.08	84.55
588568	71	59	83.10	62.23	64.15	65.69
588575	16	42	38.10	54.33	80.21	63.02
588598	45	53	84.91	85.27	88.09	85.26
588599	46	55	83.64	79.40	85.72	81.23
588600	47	54	87.04	90.95	92.27	90.36
588601	48	53	90.57	88.10	89.33	86.85
588605	35	32	91.43	77.06	74.30	80.33
588607	39	28	71.79	72.89	72.22	79.79
588609	43	24	55.81	77.78	73.23	83.91
588611	29	38	76.32	77.14	74.62	74.37
588632	28	23	82.14	100.00	100.00	100.00
588647	49	70	70.00	75.73	78.25	75.89
588678	23	40	57.50	69.33	76.52	66.97
588679	21	42	50.00	93.33	76.51	74.27
588680	26	37	70.27	73.14	80.98	73.64
588681	18	213	8.45	90.00	77.29	72.00
588688	17	14	82.35	86.00	83.46	86.00
588715	23	13	56.52	62.00	65.32	69.86
588763	183	190	96.32	74.54	71.00	74.50
588783	14	11	78.57	71.67	67.33	77.77
602129	12	22	54.55	56.67	73.89	62.63

602166	119	110	92.44	56.22	57.47	60.23
602176	11	9	81.82	86.67	82.39	82.29
602181	82	148	55.41	69.54	66.01	58.30
602202	187	393	47.58	91.96	71.41	66.44
602214	19	36	52.78	94.29	87.95	89.59
602292	375	439	85.42	77.04	77.60	74.61
602306	8	13	61.54	63.33	74.83	70.70
602356	68	74	91.89	70.83	69.02	71.70
602357	104	34	32.69	86.10	73.86	90.58
602358	102	24	23.53	92.01	70.75	95.40
602364	89	35	39.33	86.59	74.21	89.60
602365	74	45	60.81	81.56	80.64	87.52
602372	80	86	93.02	53.59	57.91	52.58
602404	32	44	72.73	84.00	90.93	84.76
602474	53	63	84.13	68.67	75.50	71.22
602475	36	86	41.86	52.00	69.90	54.77
602476	31	66	46.97	75.24	81.56	72.68
602477	87	32	36.78	91.38	88.77	93.12
602478	28	69	40.58	80.00	74.19	67.27
623883	9	16	56.25	80.00	91.65	86.15
623885	49	57	85.96	84.79	82.00	80.86
623920	12	19	63.16	80.00	72.65	70.70
623938	55	39	70.91	81.12	81.06	84.71
623973	13	18	72.22	60.33	71.99	71.70
624016	18	22	81.82	60.33	70.83	64.81
624087	20	22	90.91	84.29	80.16	85.92
624093	79	25	31.65	97.18	93.94	97.08
624094	100	10	10.00	100.00	89.11	99.63
624251	224	285	78.60	81.35	73.89	72.50
624305	296	405	73.09	72.08	70.61	66.41
624384	22	20	90.91	60.24	56.12	61.59
624455	50	1169	4.28	87.70	74.68	68.29
624487	12	14	85.71	74.17	67.82	72.02
651565	28	22	78.57	67.28	64.48	72.08
651597	388	383	98.71	74.60	73.55	73.79
651600	163	221	73.76	73.24	68.32	61.65
651605	103	8	7.77	96.19	79.18	97.57

651675	10	7	70.00	100.00	96.61	96.55
651700	169	169	100.00	72.40	66.64	66.65
651720	133	101	75.94	85.70	80.42	81.17
651741	483	956	50.52	80.65	77.85	72.90
651791	77	503	15.31	95.71	80.30	77.28
651793	93	516	18.02	95.29	76.76	73.09
651798	12	64	18.75	50.00	68.53	50.00
**Average**			**62.46**	**79.01**	**76.53**	**76.68**
**Std**			**0.25718**	**0.11647**	**0.08984**	**0.10937**

Supplementary Material 1

Steps for building a screening model

The **Model Building** page offers several options to train a specific QSAR model based on a given high-throughput screening (HTS) biological assay (or BioAssay) from **PubChem BioAssay Database**. These options include feature generation, feature selection, class imbalance solutions, and training a machine learning model.

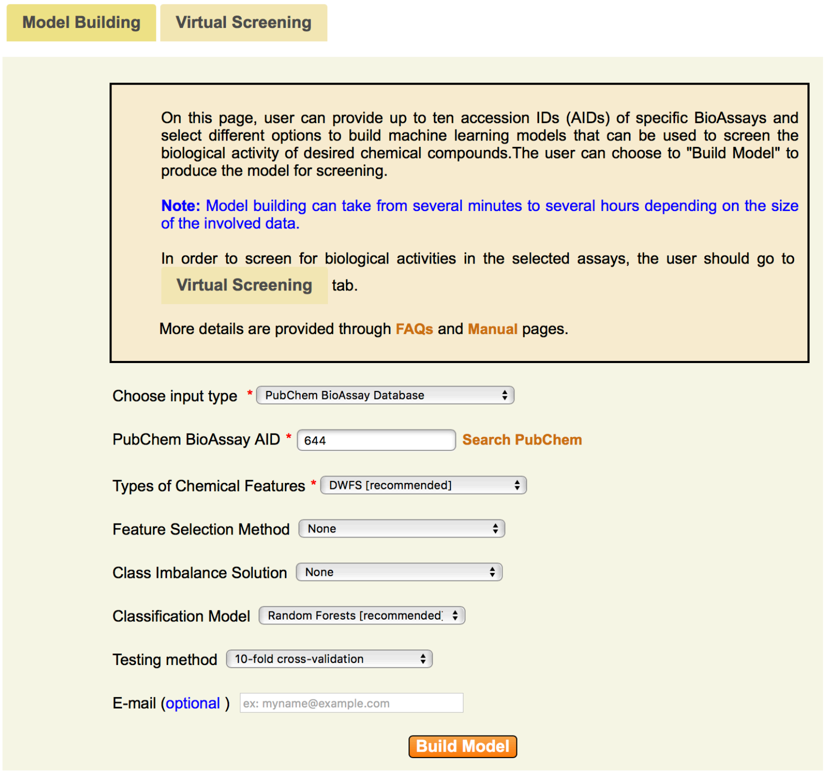

The user starts by choosing an input type. The PubChem BioAssay accession (i.e. AID) can be provided directly and the system automatically retrieves all relevant information for processing the HTS data of interest. Also, a list of AIDs can be submitted in case the user is interested in building a multilabel classification (MLC) model

where the correlation between the list of BioAssays is exploited (see Methods). Moreover, the user can provide a set of PubChem Compound accessions (i.e. CID) or SMILES with corresponding labels to build the model.

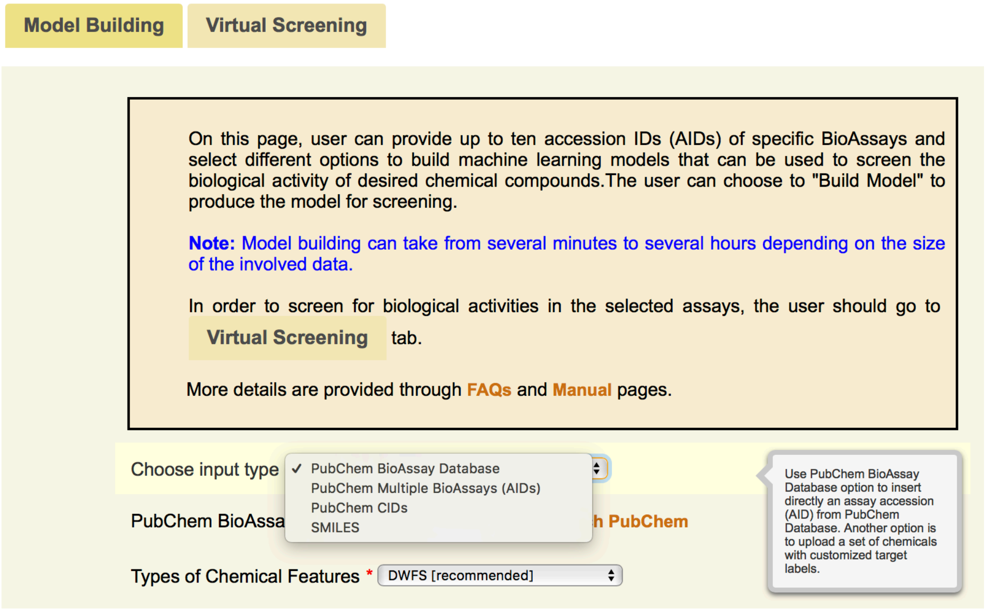

Each tested compound in the BioAssay can be represented by a different set of features. The system allows to generate a wide variety of such variables that can act as descriptors of the compounds. For references to each set of features, please refer to:

- DWFS (An optimized selection of 1064 features)
- Standard (A comprehensive list that include all the following types and other features)
- PubChem Fingerprint (881 features)
- Chemical Descriptors (37 features)
- MACCS (166 features)
- Topological Fingerprint (1024 features)

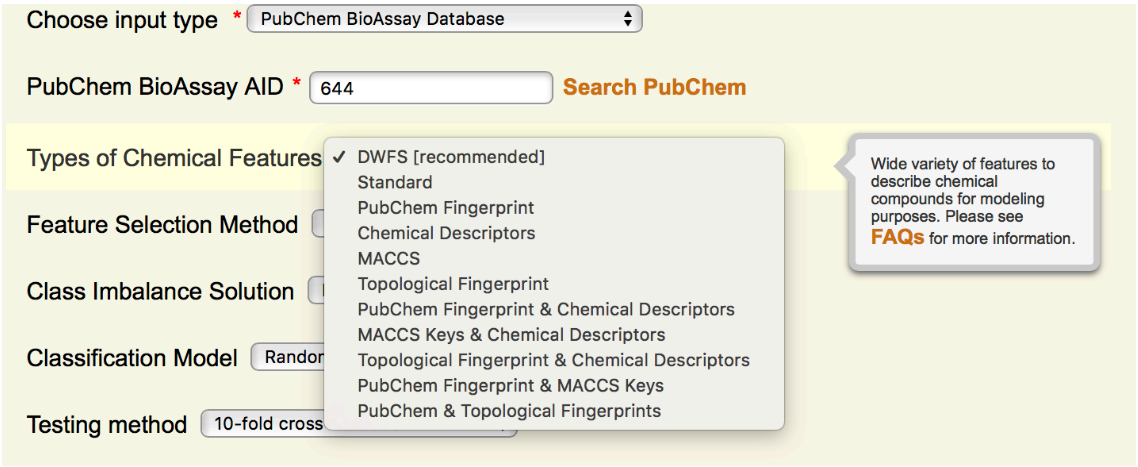

Several state-of-the-art feature selection methods are available in order to reduce the amount of input features used to derive the QSAR model. Feature selection may considerably improve the performance of the model.

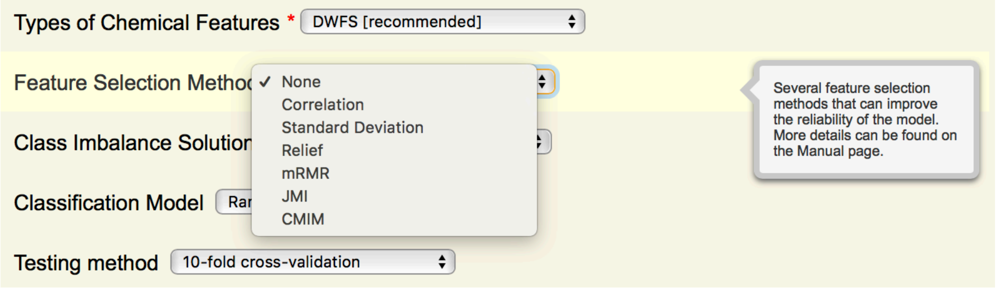

Once a particular feature selection method is chosen, the percentage of top features to be selected for deriving the QSAR model must be specified (default 10%).

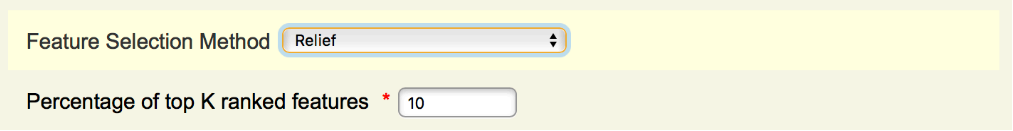

Since the HTS data, in general, face an issue of class imbalance where most of the activity labels belong to the inactive class, several state-of-the-art solutions are provided to reduce the model bias to inactive compounds. For more details about this problem in HTS data (see Results).

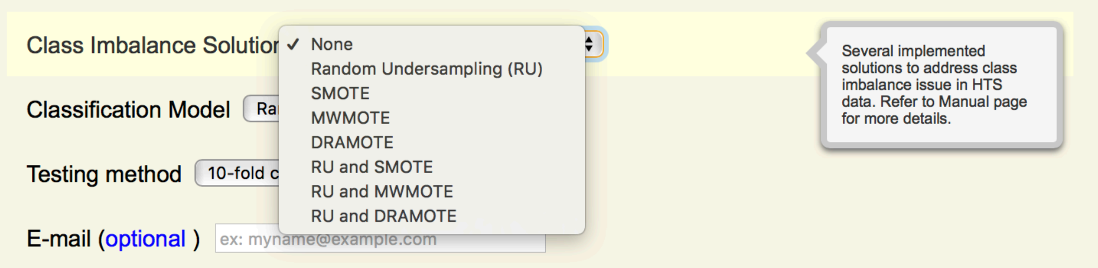

One example of the approaches to handle class imbalance is the class of synthetic minority oversampling techniques where the samples of the minority class are enriched based on interpolation. The user can decide on the percentage of the new set of minority samples to be generated (default 200%).

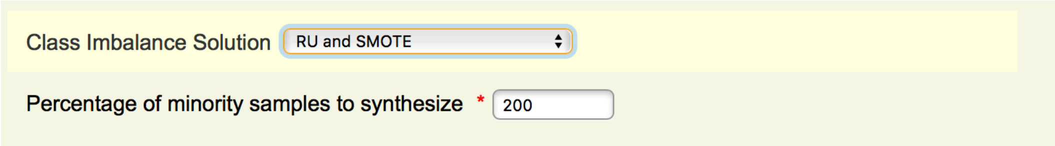

Several state-of-the-art classifiers are implemented to give the user a wide range of options in order to build a reliable QSAR model that can be used for screening and predicting biological activities of new provided chemical compounds.

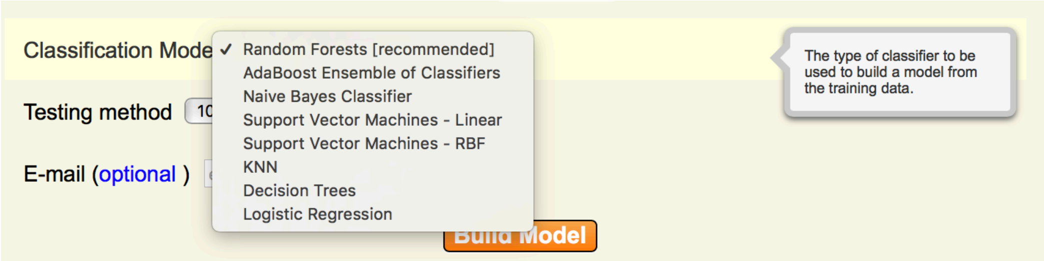

In case the user has chosen PubChem Multiple BioAssays as input type, the system DRABAL state-of-the-art solution to exploit existing correlations between the target assays.

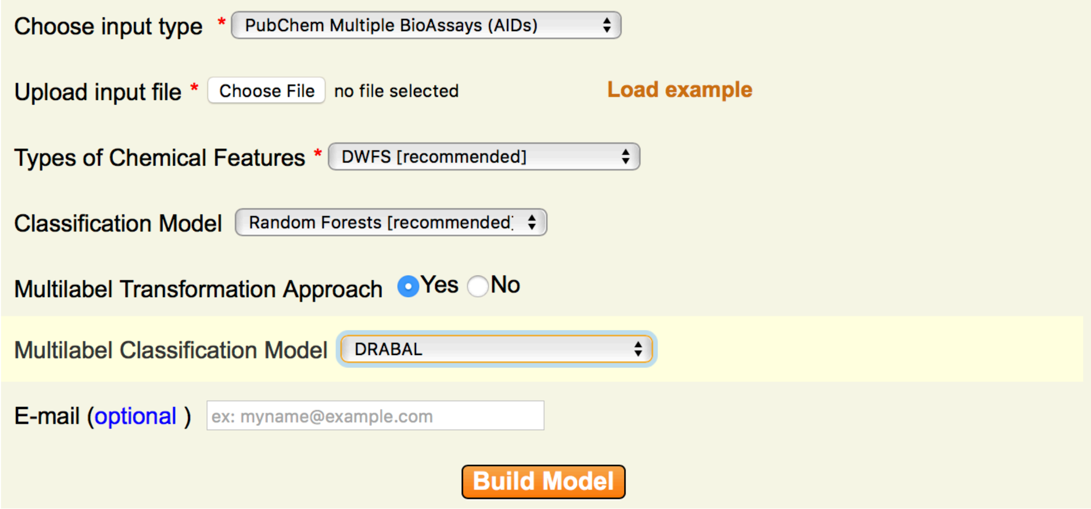

Once the user has finalized selecting the parameters and pressed on the "Build Model" button, the user will be directed to a page that displays the job status. The user is encouraged to bookmark the page in order to refer back to it for the results once the job is completed. If an email was provided, the user receives an email confirming the job submission and another email once the job is completed. In both emails, a link to the results page is provided. For more details on the results, please refer to **How the results look like?**

Once the "Model Building " job is completed, several links are available for downloading the model and data files as illustrated in the following figure.

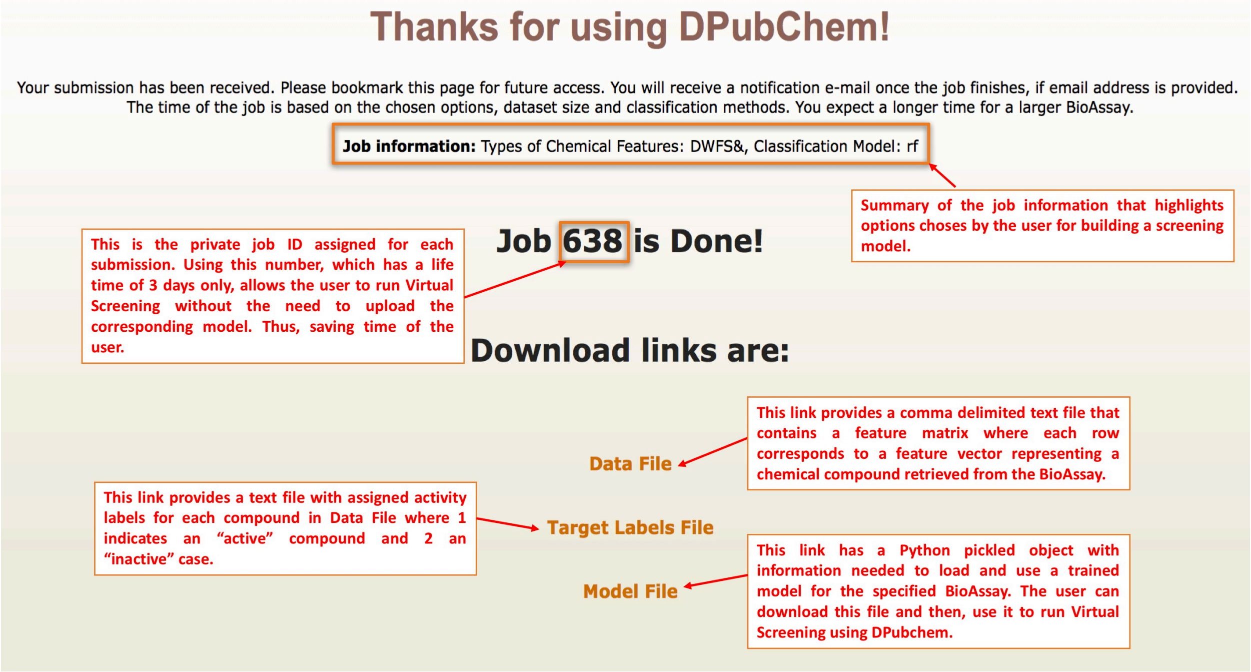

Steps for running virtual screening for drug repositioning

In order to use a model for virtual screening and predicting biological activities of new compounds, DPubChem tool provides several options to the users. A user can use the "Model Building" page to input parameters and then choose "From Model Building Page" to use these parameters in order to build a model for virtual screening. Otherwise, the user can choose from a list of pre-trained models or upload a previously trained model using our system.

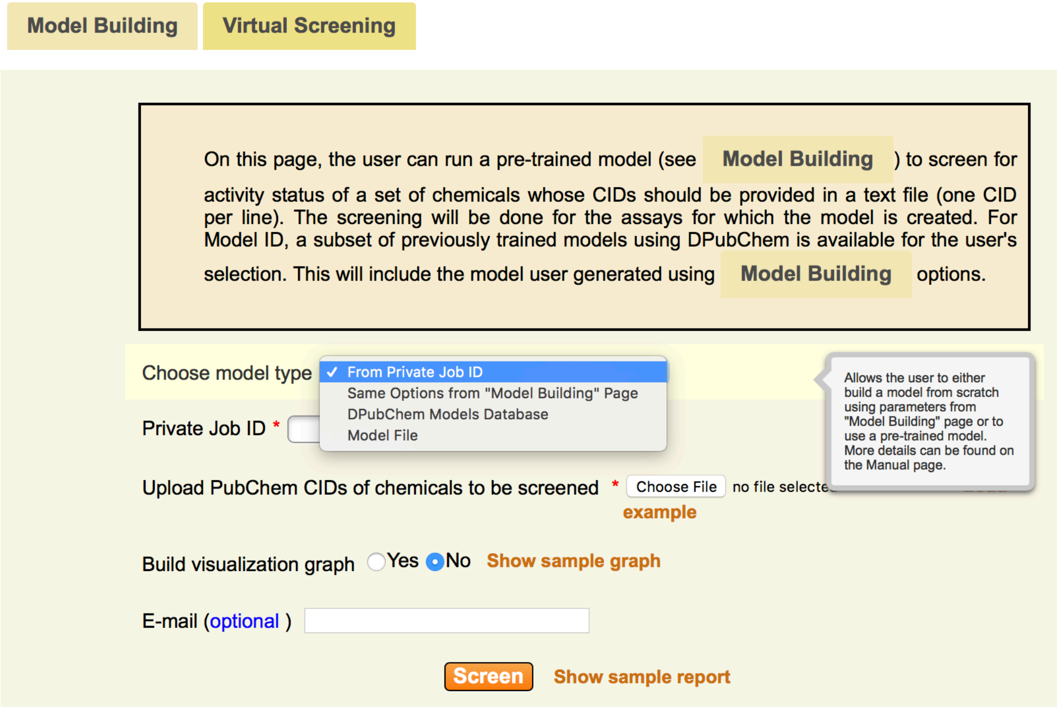

The "Choose model type" as "From Private Job ID" option, to easily use a pre-trained model. If the user has a pre-trained model, then, the corresponding job id (or private job id) can be inserted directly in the "Private Job ID" input box to avoid uploading the model. Note that this saves time of uploading large model files, facilitates the collaboration and the reproducibility of the results. However, the job id can be used up to 3 days after creating the model.

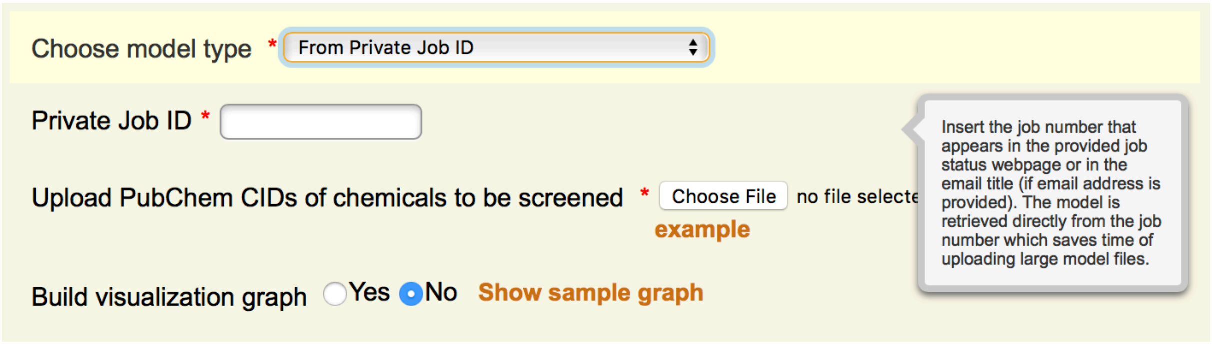

Another way to choose a pre-trained model is to select from the internally developed models and listed under "DPubChem Models Database". This option lists models for certain BioAssays that were developed internally. The list is continuously updated.

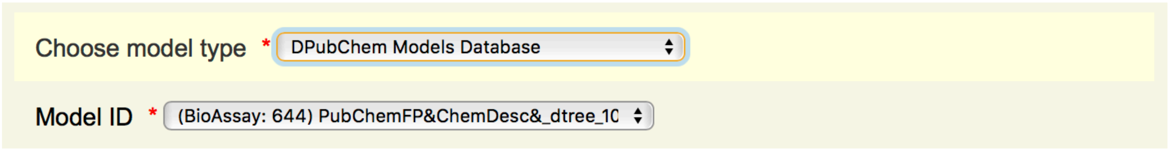

Based on the provided options, the screening process can start by clicking on the "Screen" button. One of the options to visualize output results is to build a visualization graph (interaction network). It should be noted that this graph is prepared based on a computationally intensive pipeline that measures chemical-chemical similarity, protein-protein similarity, bioassay-bioassay similarity and assign activity screening scores for chemical-bioassay interactions (see Methods).

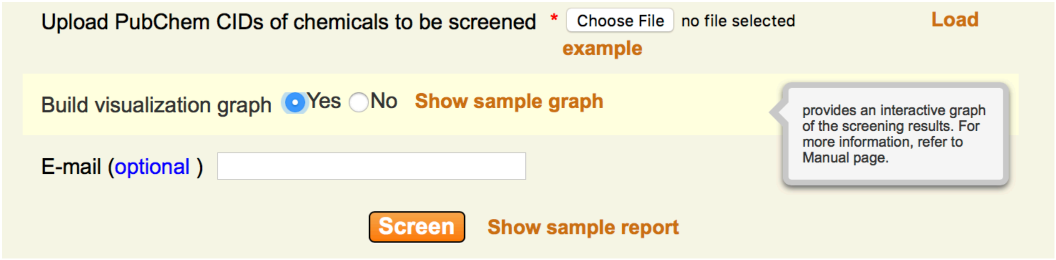

Snapshots of the output results

Once the "Virtual Screening" job is completed, several links for downloading the files are shared as illustrated in the following figure.

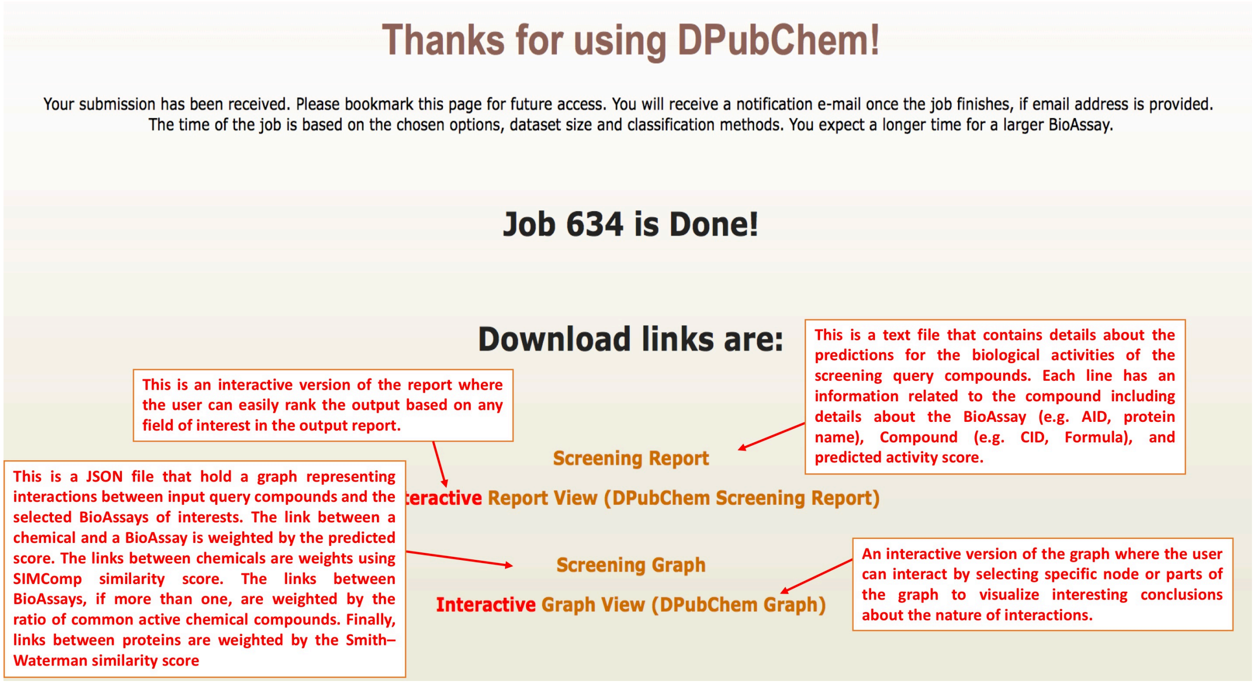

The visualization graph provides interesting insights that may highlight novel cases that otherwise would have been difficult to notice. The interaction network has a search box to easily find connections for a particular drug, chemical or protein target. Please visit this sample graph to explore the interactive graph view.

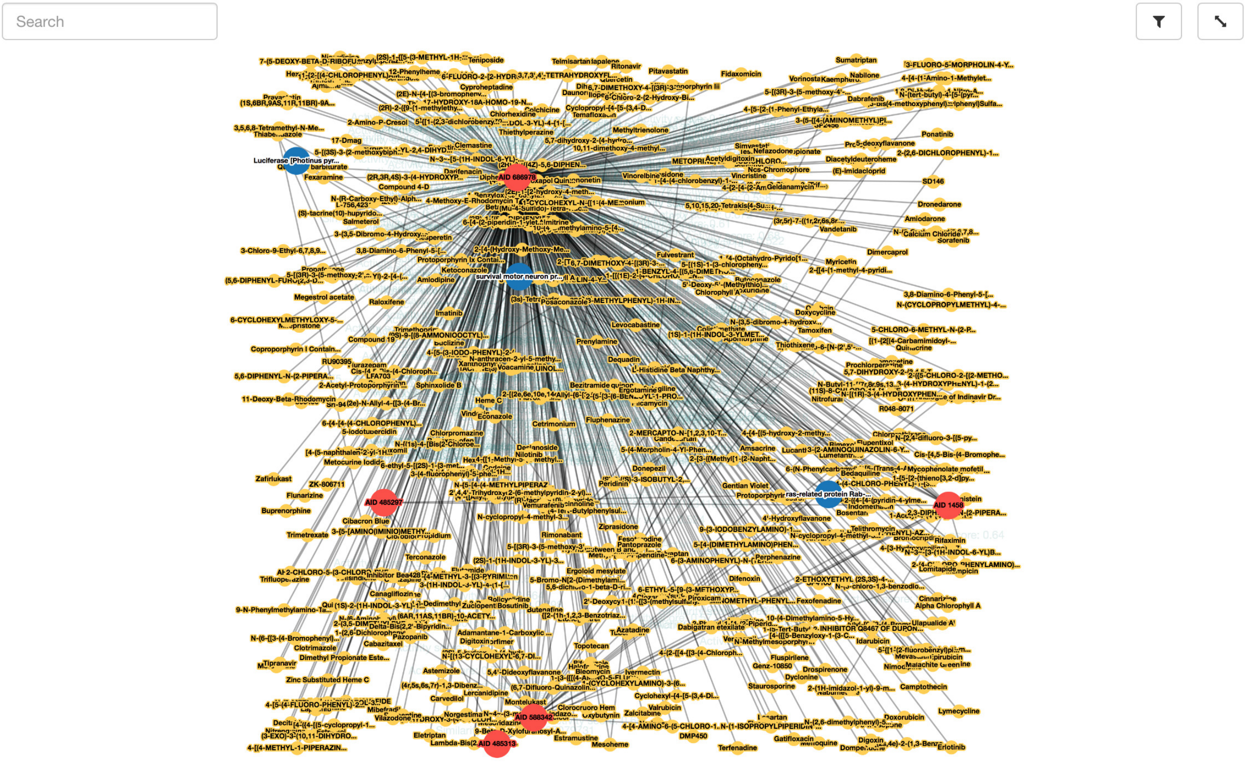

For example, the following figure highlights an interesting case where a chemical is interacting with four biological targets. This can indicate a pathway-type of relationships between the biological targets.

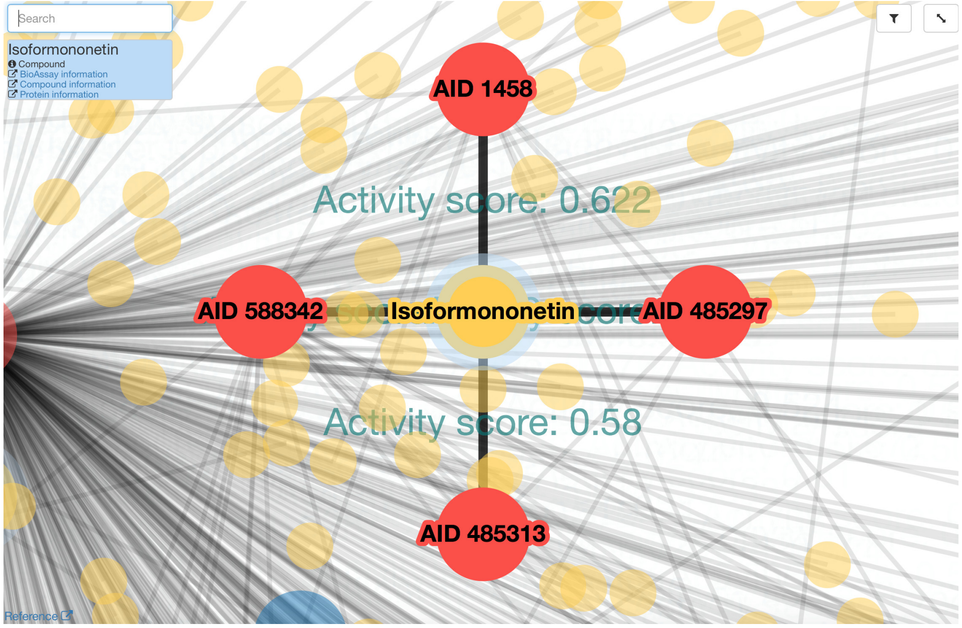

The user can use the graph filter to view only certain types of nodes. This can help in having more focus over the relationship between one group of objects like BioAssays.

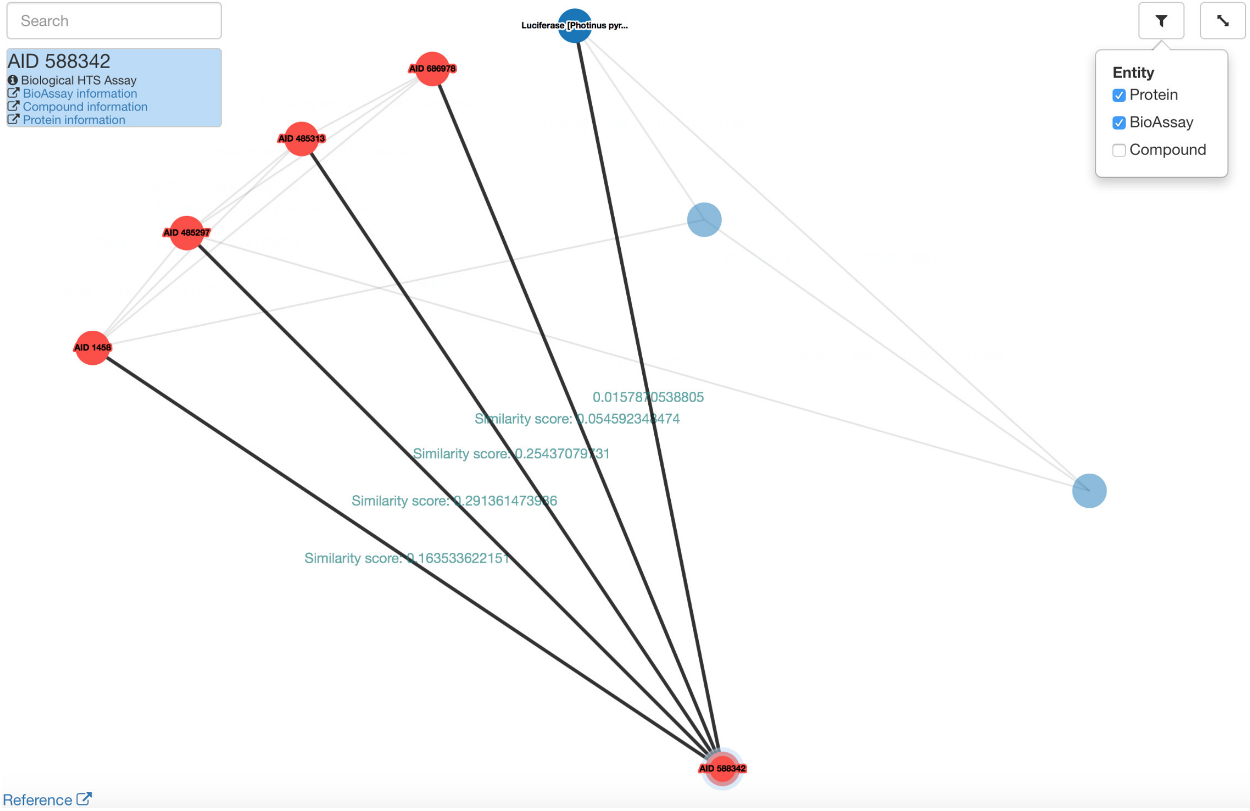

The following figure illustrates the use of graph filter to view chemical-chemical interactions. The links between chemicals are weighted by their structure similarities, which are generated using SIMComp program (see Methods).

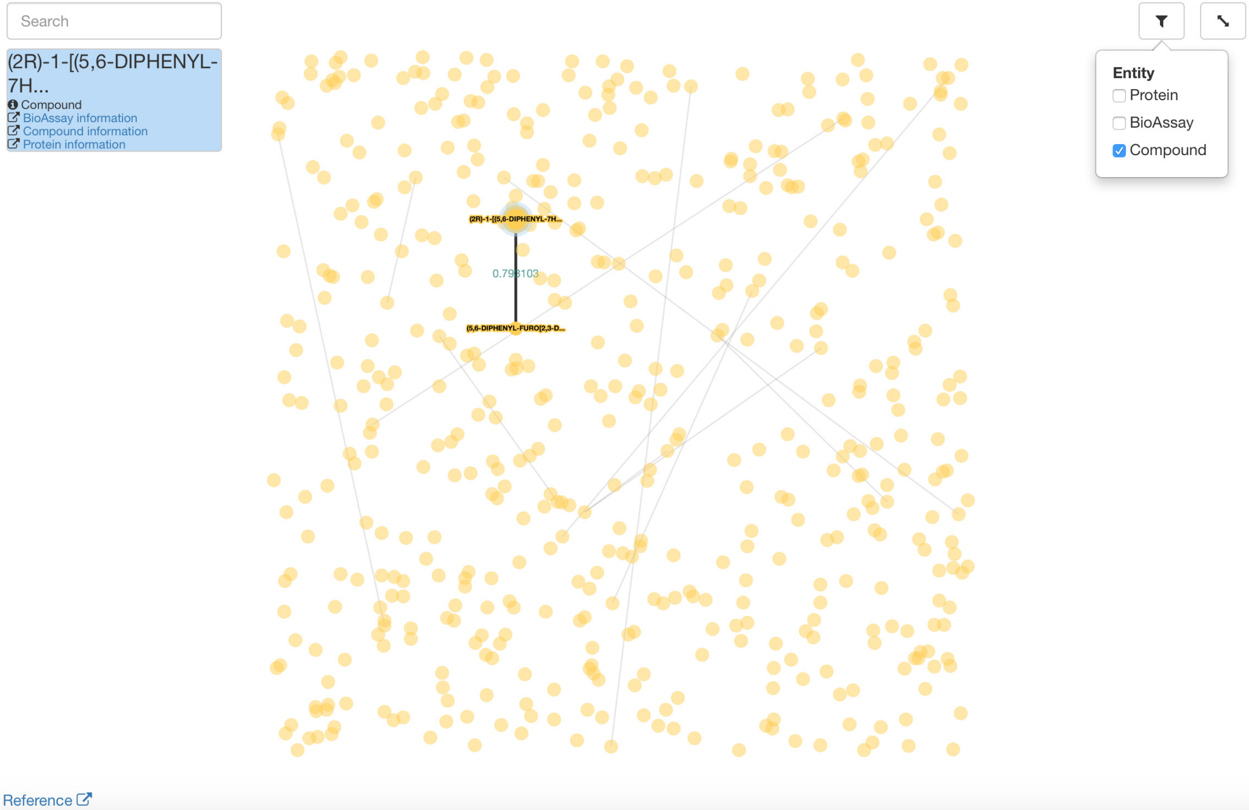

Finally, the following figure shows the output screening results provided to the user. Using this table, a user can easily rank results based on any field of interest. Please visit this **sample report** to explore the interactive results report view.

BioAssay AID	Protein ID	Protein Name	Compound CID	DrugBank ID	Drug Name	Chemical Formula	Activity Label	Activity Score
686978	79154014	TDP1 protein [Homo sapiens]	441207	DB01396	Digitoxin	C41H64O13	Active	0.978
686978	79154014	TDP1 protein [Homo sapiens]	36314	DB01229	Paclitaxel	C47H51NO14	Active	0.946
686978	79154014	TDP1 protein [Homo sapiens]	54454	DB00641	Simvastatin	C25H38O5	Active	0.926
686978	79154014	TDP1 protein [Homo sapiens]	23724511	DB00444	Teniposide	C32H32O13S	Active	0.902
686978	79154014	TDP1 protein [Homo sapiens]	5447130	DB00336	Nitrofural	C6H6N4O4	Active	0.838
686978	79154014	TDP1 protein [Homo sapiens]	3396	DB04842	Fluspirilene	C29H31F2N3O	Active	0.836
686978	79154014	TDP1 protein [Homo sapiens]	2351	DB01244	Bepridil	C24H34N2O	Active	0.766
